# PhSeZnCl in the Synthesis of Steroidal β-Hydroxy-Phenylselenides Having Antibacterial Activity

**DOI:** 10.3390/ijms20092121

**Published:** 2019-04-29

**Authors:** Izabella Jastrzebska, Stefano Mellea, Valerio Salerno, Pawel Adam Grzes, Leszek Siergiejczyk, Katarzyna Niemirowicz-Laskowska, Robert Bucki, Bonifacio Monti, Claudio Santi

**Affiliations:** 1 Institute of Chemistry, University of Białystok, ul. Ciołkowskiego 1K, 15-245 Białystok, Poland; stefano.mellea@gmail.com (S.M.); vale_1192@hotmail.it (V.S.); felolix@gmail.com (P.A.G.); nmrbial@uwb.edu.pl (L.S.); 2 Department of Microbiological and Nanobiomedical Engineering, Medical University of Białystok, ul. Mickiewicza 2C, 15-222 Bialystok, Poland; katia146@wp.pl (K.N.-L.); buckirobert@gmail.com (R.B.); 3 Group of Catalysis and Organic Green Chemistry–Department of Pharmaceutical Sciences, University of Perugia, Via del Liceo 1, 06132 Perugia, Italy; bonifaciomonti@gmail.com

**Keywords:** steroids, selenium, antibacterial, antibiofilm

## Abstract

We report here the reaction of in situ prepared PhSeZnCl with steroid derivatives having an epoxide as an electrophilic functionalization. The corresponding ring-opening reaction resulted to be regio- and stereoselective affording to novel phenylselenium-substituted steroids. Assessment of their antibacterial properties against multidrug-resistant bacteria, such as *Pseudomonas aeruginosa* Xen 5 strain, indicates an interesting bactericidal activity and their ability to prevent bacterial biofilm formation.

## 1. Introduction

The last few decades have seen a growing interest in the synthesis of organoselenium compounds due to some promising biological activities, reported in the recent literature [1,2,3]. Selenium is present in mammalian organisms in the form of selenoenzymes, embedded in the proteinogenic amino acid selenocysteine. All the selenoproteins demonstrated redox properties and have a crucial role in the redox modulation as well as in the control of the redox homeostasis of the living systems, playing also a role in the cell’s protection against the oxidative stress [4]. The prooxidant activity of organoselenium compounds was recently described as an interesting property to be directed toward specific targets developing antibacterial [5], antiviral [6], and antifungal agents [7], hormetines [8], and enzyme inhibitors [9]. Furthermore, direct and indirect interferences with the redox homeostasis and the redox signaling can produce an anticancer effect affecting the differentiation, proliferation, senescence and death pathways in the cells [10].

Even if many physiological and pathological mechanisms involving organoselenium derivatives need to be clarified, the recent introduction of Ebselen^®^ and Ebselen-like compounds in clinical trials for the treatment of diabetes complications and non-small cell lung cancer, further demonstrate the current interest in study these derivatives for medical purposes [11,12].

**Figure 1:**
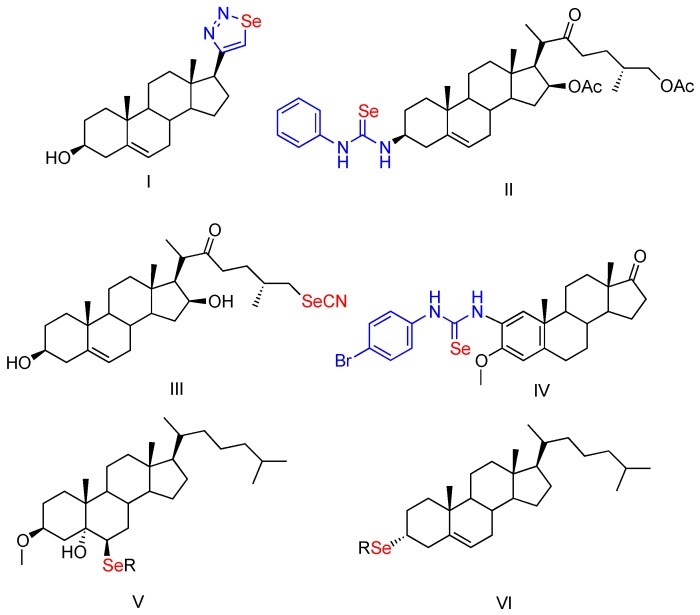
Some representative selenosteroids with antiproliferative and prooxidant activity. Selenium is directly bonded to the steroid (III, V and VI) selenium is imbedded in a different group (in blure) linked to the steroid (I, II, IV).

Till now, few examples of selenosteroids were reported in the literature and they were tested and proved to be interesting antiproliferative agents following a prooxidant mechanism. Some representative examples are reported in Figure 1 [13,14,15], selenium can be embedded as selenourea, selenocyanate or alkyl/aryl selenide directly introduced in C-3 or C-6 through the S_N_2 reaction of a selenolates with a tosylate (VI) or an epoxide (V) affording, in this letter case to a β-hydroxyselenide [16,17].

During the last ten years, some of us deeply investigated the use of nucleophilic selenium reagents, in the form of zinc selenolates, for the functionalization of electrophilic organic substrates [18]. Among these reagents the PhSeZnCl, easily prepared through the oxidative insertion of elemental zinc into Se-halogen bound of PhSeCl, it was the first bench stable organic selenolate [19], it is nowadays commercially available, and someone recently named as *Santi’s* reagent [20].

These reagents demonstrate a broad range of applicability showing in several cases a strong rate acceleration when the reaction is performed in “on water” conditions [21,22,23].

In addition, its use was recently described also in the ring opening reactions of aziridines [24] and epoxides for the optimization of a total synthesis [25] as well as in the functionalization of preformed polymers [26], demonstrating the effective applicability of the method also starting from polyfunctionalized substrates. The ring opening of epoxide with PhSeZnCl is generally highly regioselective and the presence of the zinc as Lewis acid was demonstrated to be in some cases important for the regioselective control of the reaction [19].

## 2. Results and Discussion

### 2.1. Chemistry

In the present work, we explored the use of PhSeZnCl for the functionalization of different steroid derivatives having an epoxide as an electrophilic reactive center, in order to obtain new molecular selenosteroids as prototypes for the investigation of the antibacterial activity. The optimization of the reaction conditions was performed starting from the epoxide **1** as a 4:1 mixture of the α and β isomer, respectively.

**Scheme 1:**
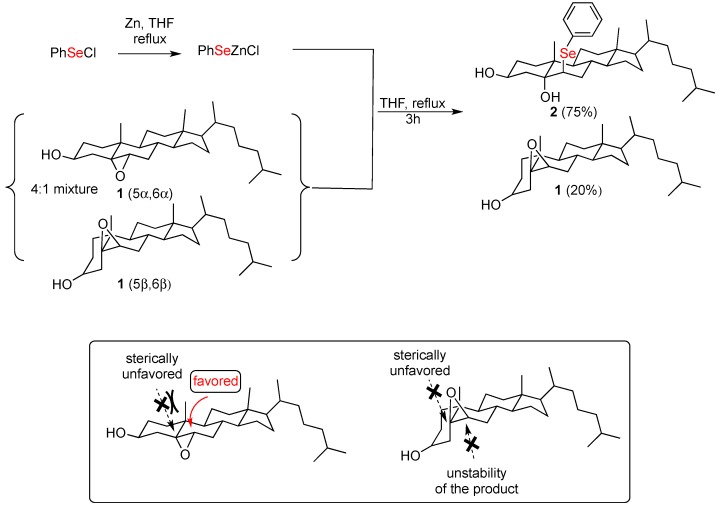
Reaction of 3β-hydroxy-5α,6α-epoxycholestane (**1**) with in situ formed PhSeZnCl.

The reaction of 5,6-epoxycholestane **1** with solid PhSeZnCl was firstly investigated using the conditions reported for the opening of epoxide both in water suspension and THF solution at room temperature [19] but these procedures did not afford the desired product. Differently, when the reagent was prepared in situ in THF and the epoxide **1** was refluxed for 3 h with the reagent the β-hydroxyselenide **2** was obtained in 60% yield (75% on converted material), as depicted in Scheme 1. The nucleophilic attack to the epoxide ring occurs exclusively on the less sterically hindered carbon (C6) due to the presence of axial C-19 methyl group, indicating that an S_N_2 mechanism is involved in the process and affording the corresponding *trans*-hydroxy selenides **2**. Only the *trans* hydroxyl selenide **2** was observed and the unreacted 5β,6β-epoxide was quantitatively recovered after chromatographic purification. The reason of the non-reactivity of 5β,6β-epoxide with nucleophilic reagents was recently explained and deal with the unfavorable formation of a constrained structure that should arise from the *trans*-diaxial opening at C6 with the hydroxyl at C5 in the *syn* position with regard to the C19 [27]. A separate experiment with epoxycholestane **1** and (PhSe)_2_ in the presence of NaBH_4_ was performed. After 3 h, TLC monitoring showed the consumption of a part of the starting material **1**. On the basis of ^1^H NMR spectral analysis, it was established that only the α-epoxide **1** reacted while the β-isomer of **1** remained intact. Moreover, after 3 h of the reaction, 3β-hydroxy-5α,6α-epoxycholestane (20%) was still present in the reaction mixture.

Compound **2** was fully characterized by NMR, IR and MS spectroscopies and the collected data correspond to those previously reported by Rodrigues et al. [16].

In the optimized protocol, the zinc (1.0 equivalent) activated by treatment with HCl 10%, was added to the solution of PhSeCl (1.0 equivalent) in anhydrous THF and heated at the reflux temperature till the discoloration of the solution that is assumed to be indicative for the formation in situ of PhSeZnCl. At this point, the substrate **1** (1.0 equiv) dissolved in THF was added. The reaction was refluxed for an additional 3 h and monitored by thin-layer chromatography (TLC).

**Figure 2:**
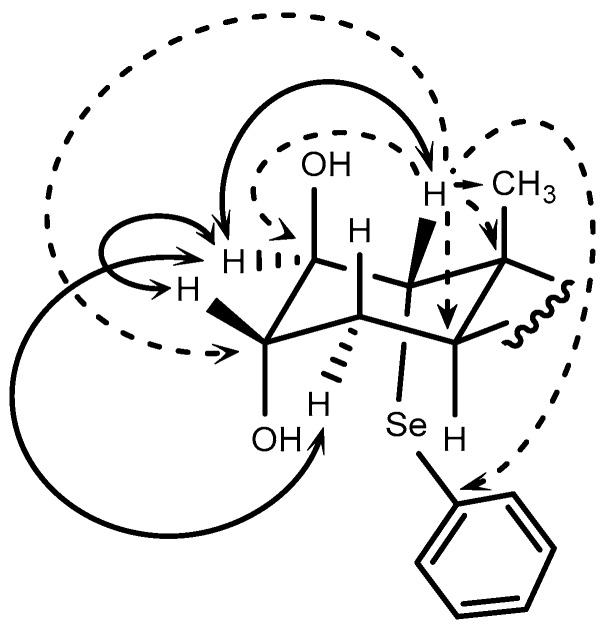
Relevant data from COrrelation SpectrocpY (COSY and heteronuclear multiple bond correlation (HMBC) experiments for the structure of the compound **9** (COSY correlations – plain line, HMBC correlations–dashed line, -- means no relation).

Using the above-described conditions, the scope of epoxy substrates **1, 3, 5, 6** and **8** was explored. The results are collected in Table 1. Interestingly the mild conditions of the procedure resulted compatible with the sensitive spiroacetal system [28] in **3** and the sterically hindered epoxide **5** [29] was totally unreactive. The reactions of 1,2-epoxysteroids **6** and **8** with the in situ generated organoselenium reagent gave 1β,3β-dihydroxy-2β-phenylselenylcholestane (**7**) and 2β,3α-dihydroxy-1α-phenylselenylcholestane (**9**), respectively. For the accurate determination of the stereochemistry of the centers formed in the C-1 and C-2 positions, two-dimensional NMR experiments were performed (COrrelation SpectrocpY (COSY), heteronuclear single quantum correlation (HSQC), and heteronuclear multiple bond correlation (HMBC)) in 400 MHz instrument. For compound **7**, the coupling system inferred from the correlation spectra indicates that phenylselenyl group is bonded to C-2, since 2-H is coupled to both adjacent 1-H and 3-H. In addition, the 3-H signal has the width at half-height of 24 Hz, which clearly indicates that it is axial, and therefore, 2-H must be equatorial because its width at half-height is small (10 Hz). The downfield shift of C-1 (78.4) shows that the configuration of the OH group is β (axial), and therefore, 1-H is equatorial. The correlation pattern inferred from COSY, HSQC, and HMBC spectra of compound **9** allows us to state that phenylselenyl group is bonded to C-1. Proton 3-H clearly has configuration β (equatorial) since its width at half-height is 8 Hz. In turn, 2-H has configuration α (equatorial) since long range coupling to 4-H_eq_ is observed in COSY spectrum filling the marks of the H-C-C-C-H W-shape structure (Figure 2) [30].

**Scheme 2:**
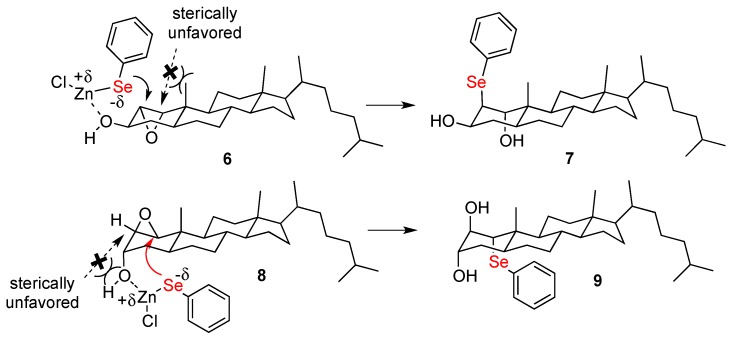
The proposed mechanism for ring opening of **6** and **8** according to a pseudo-axial ring opening approach.

Based on these spectroscopic evidence, stereogenic centers at C-1 and C-2 have the absolute configuration depicted in structure **7** and **9**, and a plausible mechanism for their formation starting from 1,2-epoxysteroids **6** and **8** with PhSeZnCl is proposed in Scheme 2. In both cases, the nucleophilic attack proceeds in order to minimize the steric hindrance of the approaching selenium reagent with the substituents, following a pseudo-axial attack according to the Fürst-Plattner rule [31]. Furthermore, the interaction of zinc with the hydroxyl group at C-3 could increase the selenium nucleophilicity and cooperate on driving the observed regioselectivity. Steric factors and the lack of a suitable pseudo-axial approach resulted to be particularly detrimental in the reactivity of PhSeZnCl with the epoxy-steroids and could explain all the observed failures.

### 2.2. Biological Activity

A further purpose of this study was to investigate the biological properties of the prepared selenosteroids. The search for new compounds that exhibit antimicrobial properties is a big challenge and an emerging need in modern medicine, especially in the context of the growing number of infections caused by multidrug-resistant bacteria. Furthermore, the antibacterial activity against the biofilm formation of *Pseudomonas aeruginosa*, which usually causes opportunistic infections, particularly in immunocompromised patients, can be considered a promising characteristic for the development of a new class of antibiotics [32].

**Figure 3:**
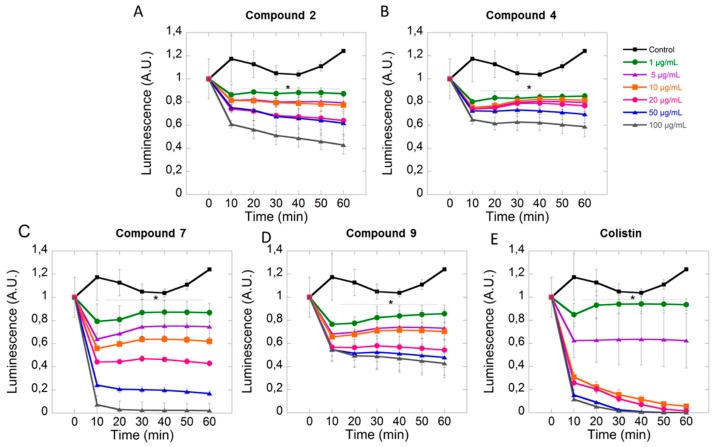
Phenylselenium-substituted steroids decrease the metabolic activity of MDR *Pseudomonas aeruginosa* strain. Panels **A**–**D** show activity of tested agents against planktonic form of *P. aeruginosa* Xen 5 in comparison to colistin (**E**). Statistical significance for the samples treated by tested compounds compared to control was marked by (*), *p* ≤ 0.05. Results from 3 measurements ±SD.

The changes of *Pseudomonas aeruginosa*, Xen 5 luminescence provide an easy way to assess bacteria cell viability and metabolic function. [33] Accordingly, Figure 3A–E shows that all tested agents affect the viability of employed bacteria strain. However, activity of compound **7** was stronger when compared to other tested agents. More than 95% of decrease in *P. aeruginosa* Xen 5 chemiluminescence, was observed after 10 min of incubation when highest dose of **7** was tested. This inhibitory effect is comparable to activity of standard antibiotic agents used in concentrations corresponding to a 100-fold increase of MIC value (Figure 3E). The obtained results suggest that the position of phenylselenyl group might affect and modulate the antimicrobial activity. Additionally, the activity of synthesized compounds, was compared to that of colistin, currently used in the treatment of *P. aeruginosa* infections. Interestingly, it was observed that the use of colistin at a dose corresponding to 1xMIC value does not affect the metabolic function of *P. aeruginosa* infections caused by MDR strains. In turn, application of 5-fold MIC concentration disturbs the cell function at 60%.

**Figure 4:**
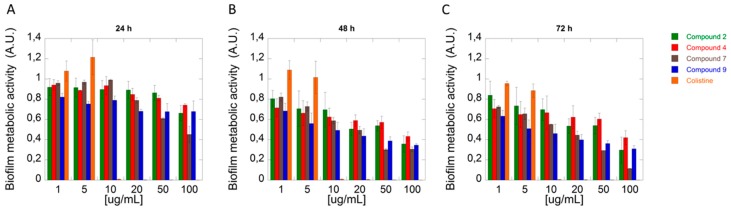
Anti-biofilm activity of phenylselenium-substituted steroids against *P. aeruginosa* strain. Ability of phenylselenium-substituted steroids to prevent biofilm-formation of *P. aeruginosa* after 24, 48 and 72 h. Results from 3 measurements ±SD.

In a different set of experiments, luminometric measurements was applied to determine the ability of the tested phenylselenium-substituted steroids on preventing bacterial biofilm formation. Figure 4A–C illustrates that the tested agents are able to inhibit biofilm formation and effectively kill bacteria embedded into the biofilm matrix in time and dose-dependent manners. However, after 24 h, in the case of compound **7**, high doses (>50 μg/mL) were required to obtain ~50% inhibition of biofilm formation. In the case of mature biofilm, formed after 48 and 72 h treatment with the tested agents (> 20 μg/mL), decreases biofilm viability by ~50%, for compound **2** and **4** respectively, and ~ 90% for compound **7**. In the case of colistin, their use in 1 and 5- fold MIC was insufficient to restrict biofilm metabolic activity. Therefore, the observed proprieties of the tested agents might help in developing of effective strategies against *Pseudomonas aeruginosa* biofilm formation, which is directly associated with hospital infections via the colonization of medical devices, as well as a major cause of the recurrence and chronic infections, such as pneumonia in cystic fibrosis patients. Due to amphipathic nature of tested phenylselenium-substituted steroids and their similarity to ceragenins, which possess a broad spectrum of antimicrobial activity, further studies are needed to determine a full antimicrobial spectrum of the tested agents and to establish their mode of action [34].

**Figure 5:**
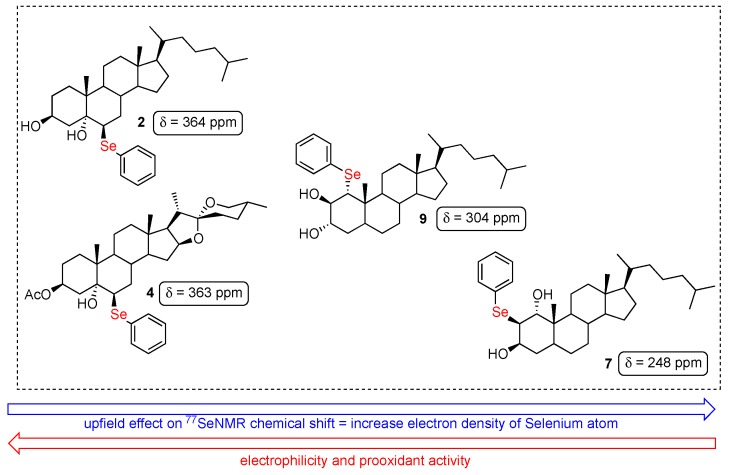
^77^SeNMR Chemical shift as parameter to predict biological aspects.

The analysis of ^77^Se-NMR chemical shift of **2**,**4**,**7** and **9** (reported in Figure 5) afforded interesting consideration. In compounds **7** and **9** the presence of hydroxyl groups in a suitable position to establish a non-bonding interaction with the selenium atom produce an evident upfield of the chemical shift (−60 ppm for **9**; −116 ppm for **7** respect **2** and **4**). This correspond to a higher electron density on the selenium atom of 7 respect to the other derivatives and, consequently, a lower electrophilicity to which correspond a reduced prooxidant activity and, reasonably, a lower general toxicity.

**Scheme 3:**
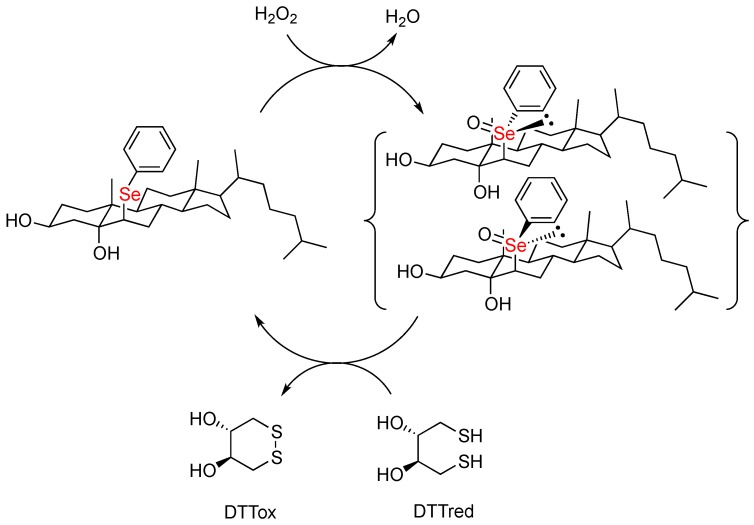
Proposed redox cycle of 3β,5α-dihydroxy-6β-phenylselenylcholestane (**2**).

Considering that Rodrigues et al. reported that derivatives similar to **2** are characterized by an important prooxidant activity we explored the redox behavior of **2** as well as its GPx-like activity using the ^77^Se-NMR and NMR-DTT coupled test [16,17]. Initially, we investigated by ^77^Se-NMR spectroscopy, the actual intermediates involved in GPx-like cycle. With the addition of hydrogen peroxide (5 eq), we observed the formation of a couple of diastereomeric selenoxides (δ ^77^Se-NMR in CD_3_OD: 901 and 866 ppm), that can be readily reduced to selenide by the addition of a stoichiometric amount (5 eq) of reduced dithiotreitol (DTTred). The experiment was performed directly into the NMR tube (Scheme 3). The kinetics of the peroxide reduction catalyzed by **2** was measured studying via ^1^H-NMR the oxidation of DTT. This process resulted particularly slow producing, after 19 h, only 22% of oxidized DTT. This value is particularly low respect those reported in literature for other GPx mimetics and we believe that it could be due to the steric hindrance around the selenium atom of selenoxide, that prevented the fast attack of the thiol and consequently the reduction of the selenoxide.

## 3. Materials and Methods

### 3.1. Chemistry

#### 3.1.1. General Methods

Reagent-grade chemicals were purchased and used as received. Methylene chloride was freshly distilled. Flash column chromatography and flash chromatography were performed with silica gel, pore size 40A (70–230 mesh), unless otherwise stated. All reactions were monitored by TLC on silica gel plates 60 F_254_. ^1^H (400 MHz) and ^13^C NMR (100 MHz) spectra for all compounds were recorded at ambient temperature and were referenced to TMS (0.0 ppm) and CDCl_3_ (77.0 ppm), respectively, unless otherwise noted. NMR resonance multiplicities were reported using the following abbreviations: b = broad, s = singlet, d = doublet, t = triplet, q = quartet, and m = multiplet; coupling constants *J* were reported in Hz. IR spectra were obtained in a CHCl_3_ solution with an FT-IR spectrometer, and data are reported in cm^−1^. Melting points were determined by a Kofler bench (Boetius type) apparatus and are uncorrected. (Spectra of the synthetized compounds are collected in the Supplementary Materials)

#### 3.1.2. General Procedure

To a solution of the PhSeCl (1.0 equiv.) in anhydrous THF (1 mL/mmol) under argon atmosphere and at reflux, the activated zinc (1.0 equiv) was added. The formation of PhSeZnCl was indicated by the formation of colourless solution. Then, a solution of the starting material (1.0 equiv) in THF under argon atmosphere was added. The reaction was stirred at reflux for 3–8 h. Then THF was removed under vacuum. The yellow oil obtained was dissolved in CH_2_Cl_2_ and washed 3 times with H_2_O. The organic layers were dried with Na_2_SO_4_, filtered and the solvent removed under vacuum. The products were purified by flash chromatography.

##### β,5α-Dihydroxy-6β-Phenylselenylcholestane (**2**)

The reaction with 3β-hydroxy-5ζ,6ζ-epoxycholestane [35] (1, 4α:1β, 100 mg, 0.2 mmol) was carried out (reaction time 3 h). Silica gel column chromatography gave pure compound **2** as a white solid (69 mg; 60%) eluted with ethyl acetate/hexane 1:4, and 3β-hydroxy-5β,6β-epoxycholestane was recovered. **2**: m.p. 159–162 °C (CH_2_Cl_2_/hexane). IR ν_max_ (cm^−1^): 3554, 3407, 1064. ^1^H NMR δ 7.56 (m, 2H), 7.27 (m, 3H), 4.08 (m, 1H), 3.10 (m, 1H), 2.46 (dd, *J* = 11.2, 13.3 Hz, 1H), 1.18 (s, 3H), 0.92 (d, *J* = 6.5 Hz, 3H), 0.88 (d, *J* = 6.6 Hz, 3H), 0.87 (d, *J* = 6.6 Hz, 3H), 0.74 (s, 3H). ^13^C NMR δ 134.0 (CH × 2), 132.6 (C), 129.1 (CH × 2), 129.1 (CH), 78.1 (C), 68.1 (CH), 56.3 (CH), 55.5 (CH), 54.2 (CH), 46.1 (CH), 55.7 (CH), 45.4 (CH), 44.2 (CH_2_), 42.8 (C), 39.9 (CH_2_), 39.5 (CH_2_), 39.3 (C), 36.2 (CH_2_), 35.8 (CH), 34.5 (CH_2_), 32.6 (CH_2_), 31.3 (CH_2_), 30.8 (CH_2_), 28.2 (CH_2_), 28.0 (CH), 24.2 (CH_2_), 23.8 (CH_2_), 22.8 (CH_3_), 22.5 (CH_3_), 21.3 (CH_2_), 18.6 (CH_3_), 17.6 (CH_3_), 12.2 (CH_3_). ^77^Se NMR δ 365.8. HRMS calcd. for C_33_H_52_O_2_Se: 560.3133, found: 560.3120.

##### (25R)-5α-Hydroxy-6β-Phenylselenylspirostan-3β-ol Acetate (**4**)

The reaction with (25*R*)-5α,6α-epoxyspirostan-3β-ol acetate [36] (3, 85 mg, 0.2 mmol) was carried out (reaction time 6 h). Silica gel column chromatography gave pure compound **4** as a white solid (70 mg; 62%) eluted with ethyl acetate/hexane 5:95. Colorless crystals: m.p. 270–273 °C (CH_2_Cl_2_/hexane). IR, ν_max_ (cm^−1^) 3427, 3005, 1710, 1064. ^1^H NMR: 7.54 (m, 2H), 7.27 (m, 3H), 5.15 (m, 1H), 4.39 (m, 1H), 3.47 (dd, *J* = 2.6, 9.4 Hz, 1H), 3.37 (m, *J* = 10.9 Hz, 1H), 3.06 (m, 1H), 2.47 (dd, *J* = 11.3, 13.4 Hz), 2.05 (s, 3H), 1.20 (s, 3H), 0.97 (d, *J* = 6.9 Hz, 3H), 0.85 (s, 3H). 0.79 (d, *J* = 6.3 Hz, 3H); δ ^13^C NMR: δ 171.8 (C), 134.7 (CH × 2), 131.9 (C), 129.1 (CH × 2), 127.1 (CH), 109.2 (C), 80.7 (CH), 71.2 (CH), 66.8 (CH_2_), 62.2 (CH), 55.2 (CH), 54.0 (CH), 45.7 (CH), 40.8 (C), 40.4 (CH_2_), 39.9 (CH_2_), 39.5 (C), 36.6 (CH_2_), 33.9 (CH_2_), 32.3 (CH_2_), 31.7 (CH_2_), 31.4 (CH_2_), 30.8 (CH), 30.3 (CH), 28.8 (CH_2_), 26.6 (CH_2_), 21.4 (CH_3_), 21.0 (CH_2_), 17.5 (CH_3_), 17.1 (CH_3_), 16.6 (CH_3_), 14.5 (CH_3_). ^77^Se NMR δ 363.1. HRMS calcd. for [C_35_H_50_O_5_SeH]^+^: 631.2902, found: 631.2877.

##### 1α,3β-Dihydroxy-2β-Phenylselenylcholestane (**7**)

The reaction with 3β-hydroxy-1α,2α-epoxycholestane [37] (**6**) (80 mg; 0.2 mmol) was carried out for 2 h. Silica gel column chromatography gave pure compound **7** eluted with ethyl acetate/hexane 15:85 as oil (60 mg; 54%). IR ν_max_ (cm^−1^): 3429, 3055, 1458. ^1^H NMR δ 7.64 (m, 2H), 7.26 (m, 3H), 4.39 (s, 1H), 4.09 (m, 1H), 3.78 (t, *J* = 2.5 Hz, 1H), 2.34 (d, *J* = 11.2 Hz, 1H), 0.96 (s, 3H), 0.91 (d, *J* = 6.5 Hz, 3H), 0.87 (d, *J* = 6.6 Hz, 3H), 0.86 (d, *J* = 6.6 Hz, 3H), 0.67 (s, 3H). ^13^C NMR δ 132.8 (C), 132.7 (CH × 2), 129.2 (CH × 2), 127.3 (CH), 78.4 (CH), 67.4 (CH), 59.5 (CH), 56.3 (CH × 2), 47.7 (CH), 42.6 (C), 40.1 (C), 39.8 (CH_2_), 39.5 (CH_2_), 39.0 (CH), 36.1 (CH_2_), 35.9 (CH_2_), 35.7 (CH), 34.9 (CH), 31.5 (CH_2_), 28.2 (CH_2_), 28.0 (CH_2_), 27.9 (CH), 24.2 (CH_2_), 23.8 (CH_2_), 22.8 (CH_3_), 22.5 (CH_3_), 20.9 (CH_2_), 18.7 (CH_3_), 13.5 (CH_3_), 12.1 l(CH_3_). ^77^Se NMR δ 248.4 ppm. HRMS calcd. for C_33_H_52_O_2_Se: 560.3133, found: 560.3121.

##### 2β,3α-Dihydroxy-1α-Phenylselenylcholestane (**9**)

The reaction with 3α-hydroxy-1β,2β-epoxycholestane [38] (**8**) was carried out for 2 h. Silica gel column chromatography gave pure compound **9** eluted with ethyl acetate/hexane 1:4. as white solid (60 mg, 53%); m.p. 174–175 °C (CH_2_Cl_2_/hexane); IR ν_max_ (cm^−1^): 3565, 3351, 1437. ^1^H NMR δ, ppm 7.54 (m, 2H), 7.26 (m, 3H), 4.39 (m, 1H), 4.09 (s, 1H), 3.50 (d, *J* = 2.6 Hz, 1H), 2.47 (bs, 1H), 2.11 (bs, 1H), 1.19 (s, 3H), 0.91 (d, *J* = 6.5 Hz, 3H), 0.88 (d, *J* = 6.6 Hz, 3H), 0.87 (d, *J* = 6.6 Hz, 3H), 0.68 (s, 3H). ^13^C NMR δ 133.8 (CH × 2), 130.2 (C), 129.2 (CH × 2), 127.4 (CH), 73.9 (CH), 67.9 (CH), 57.4 (CH), 56.2 (CH), 56.1 (C), 52.4 (CH), 42.6 (C), 41.3 (CH), 39.6 (CH_2_), 39.5 (CH_2_), 36.1 (CH_2_), 35.7 (CH), 35.1 (CH), 32.9 (CH_2_), 31.5 (CH_2_), 28.7 (CH_2_), 28.2 (CH_2_), 27.9 (CH), 24.2 (CH_2_), 23.8 (CH_2_), 22.8 (CH_3_), 22.5 (CH_3_), 20.7 (CH_2_), 18.6 (CH_3_), 15.9 (CH_3_), 12.1 (CH_3_). ^77^Se NMR δ 304.5 ppm. HRMS calcd. for C_33_H_52_O_2_Se: 560.3133, found: 560.3139.

### 3.2. Biological Activity

#### 3.2.1. Antibacterial Testing

To assess the biological activities of synthesized compounds, we have chosen to determine their antibacterial activity against Gram-negative, multidrug resistant (MDR) *Pseudomonas aeruginosa* Xen 5 strain. Briefly, a bacterial culture was grown to mid-log phase at 37 °C, re-suspended in LB, and brought to 10^8^ CFU/mL. Then 100 μL of bacteria suspensions were added to tested agents at a concentration range 1–100 μg/mL. Colistin was use as a control, since it represents “last treatment option” in some infections caused by MDR *Pseudomonas aeruginosa.* Upon bacteria addition the chemiluminescence intensity were registered using Labsystems Varioscan Flash (Thermo Scientific Waltham, MA, USA) over a period of 1h.

#### 3.2.2. Anti-Biofilm Activity

The biomass of biofilm, formed by *Pseudomonas aeruginosa* Xen 5 in the presence of tested molecules, was evaluated using a luminescence technique as described previously [39]. Chemiluminescence intensity of *Pseudomonas aeruginosa* Xen 5 biomass was measured after 24, 48, and 72 h of growth. In this setting, time-dependent biofilm mass and viability was determined

#### 3.2.3. GPx-Like Activity by NMR

In a 5mm NMR tube containing 0.15 mmol of DTT^red^ and 0.015 of catalyst (**2**) dissolved in 0.55 mL of CD_3_OD, 0.15 mmol of H_2_O_2_ (30%) was added and ^1^H-NMR experiments at 200 MHz were recorded at regular interval for a period of 24 h. Ratios between DTT^red^/DTT^ox^ were determined by comparison of integrals at 2.88 ppm and 2.64 ppm, respectively.

## 4. Conclusions 

In conclusion we demonstrated that in situ formed PhSeZnCl can be used for the functionalization of steroids with selenium moieties. The obtained novel compounds were tested for their potential antibacterial properties against *Pseudomonas aeruginosa* Xen 5 strain, evidencing a promising bactericidal activity associated to a biofilm formation prevention.

## Figures and Tables

**Table 1:** Scope of the reaction

Entry	Substrate	Time (h)	Product	Yield
1	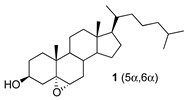	3	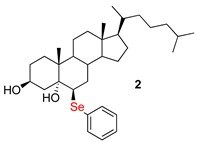	75%
2	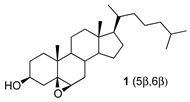	3	--	--
3	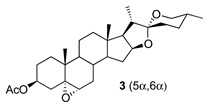	6	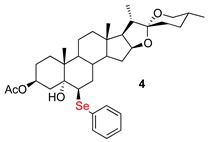	62
4	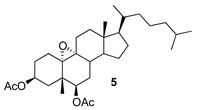	8	--	--
5	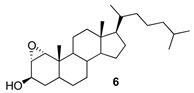	2	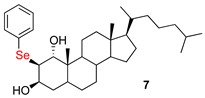	54
6	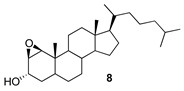	2	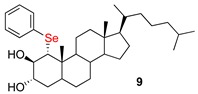	53

## Supplementary Materials

Supplementary materials can be found at https://www.mdpi.com/1422-0067/20/9/2121/s1.

## Abbreviations

COSY	COrrelation SpectrocpY
DTT	DiThioTreitol
GPx	Glutathione Peroxidase
HMBC	Heteronuclear Multiple Bond Correlation
HSQC	Heteronuclear Single Quantum Correlation
IR	InfraRed
MIC	Minimum Inhibitory Concentration
MS	Mass
NMR	Nuclear Magnetic Resonance
SD	Standard Deviation
THF	TetraHydroFuran
TLC	Thin Layer Chromatography
