# Supplementary material for: PhSeZnCl in the Synthesis of Steroidal β-Hydroxy-Phenylselenides Having Antibacterial Activity
Source: Int J Mol Sci. 2019 Apr 29;20(9):2121. doi: 10.3390/ijms20092121 (PMC6539910; doi:10.3390/ijms20092121)
Supplement: Supplementary file 1 [file ijms-20-02121-s001.pdf]

PhSeZnCl in the Synthesis of Steroidal β-Hydroxy-Phenylselenides Having Antibacterial Activity

Izabella Jastrzebska [1,*], Stefano Mellea [1], Valerio Salerno [1], Paweł Adam Grześ [1],
Leszek Siergiejczyk [1], Katarzyna Niemirowicz-Laskowska [2], Robert Bucki [2], Bonifacio Monti [3] and Claudio Santi [3,*]

[1] Institute of Chemistry, University of Białystok, ul. Ciołkowskiego 1K, 15-245 Białystok, Poland
[2] Department of Microbiological and Nanobiomedical Engineering, Medical University of Białystok, ul. Mickiewicza 2C, 15-222 Bialystok, Poland
[3] Group of Catalysis and Organic Green Chemistry–Department of Pharmaceutical Sciences, University of Perugia, Via del Liceo 1, 06132 Perugia, Italy

Supplementary contens:

1) Compound **2,** Figures S1–S6; pp.2–7
2) Compound **4**, Figures S7–S12; pp.8–13
3) Compound **7**, Figures S13–S18; pp.16–21
   Table S1. ^{1}H NMR and ^{13}C-NMR chemical shifts of compound **7** pp.14–15
4) Compound **9**, Figures S1–S24; pp.24–29
   Table S2. ^{1}H NMR and ^{13}C-NMR chemical shifts of compound **9** pp. 22–23

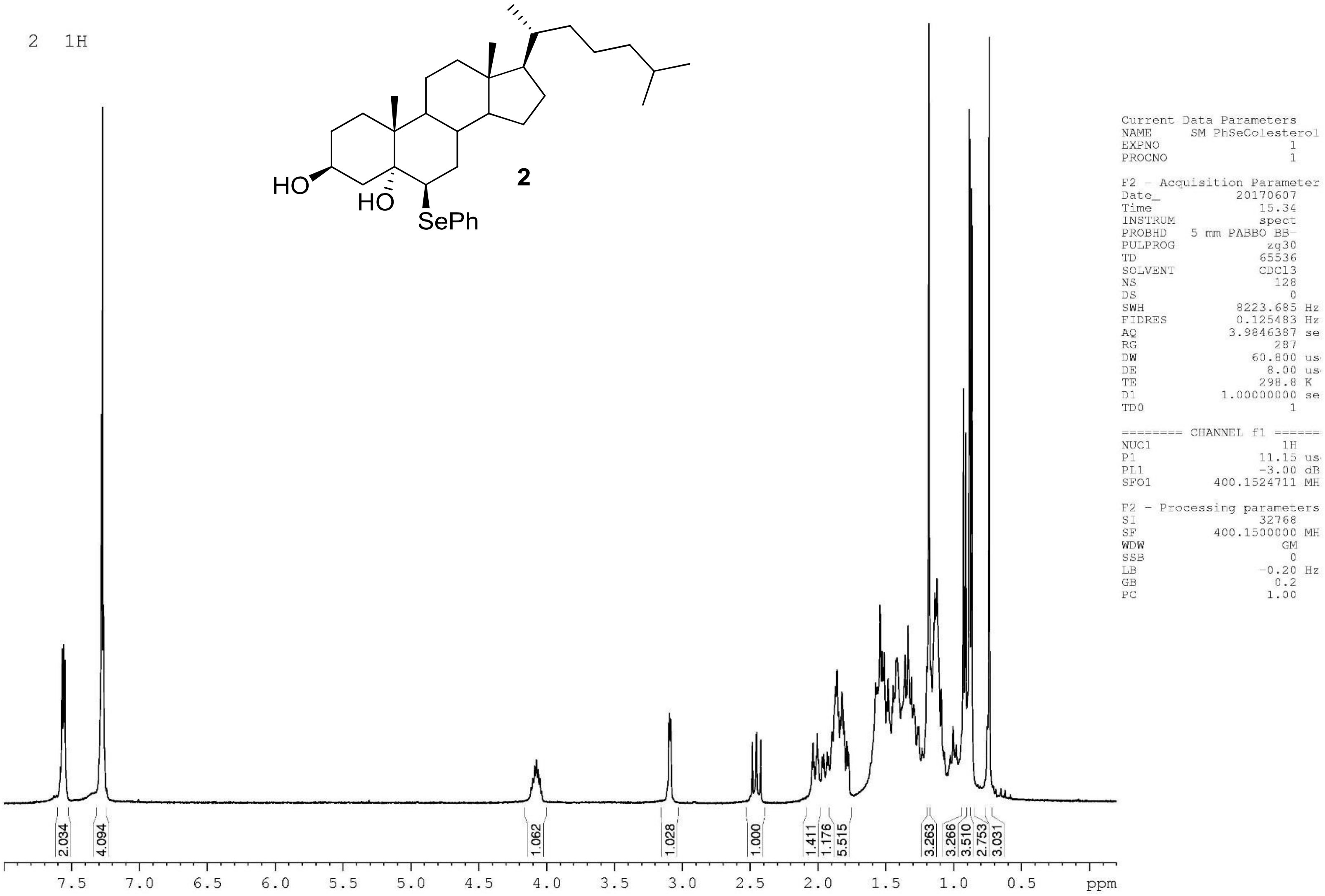

Figure S1. ^{1}H NMR spectrum of compound **2**

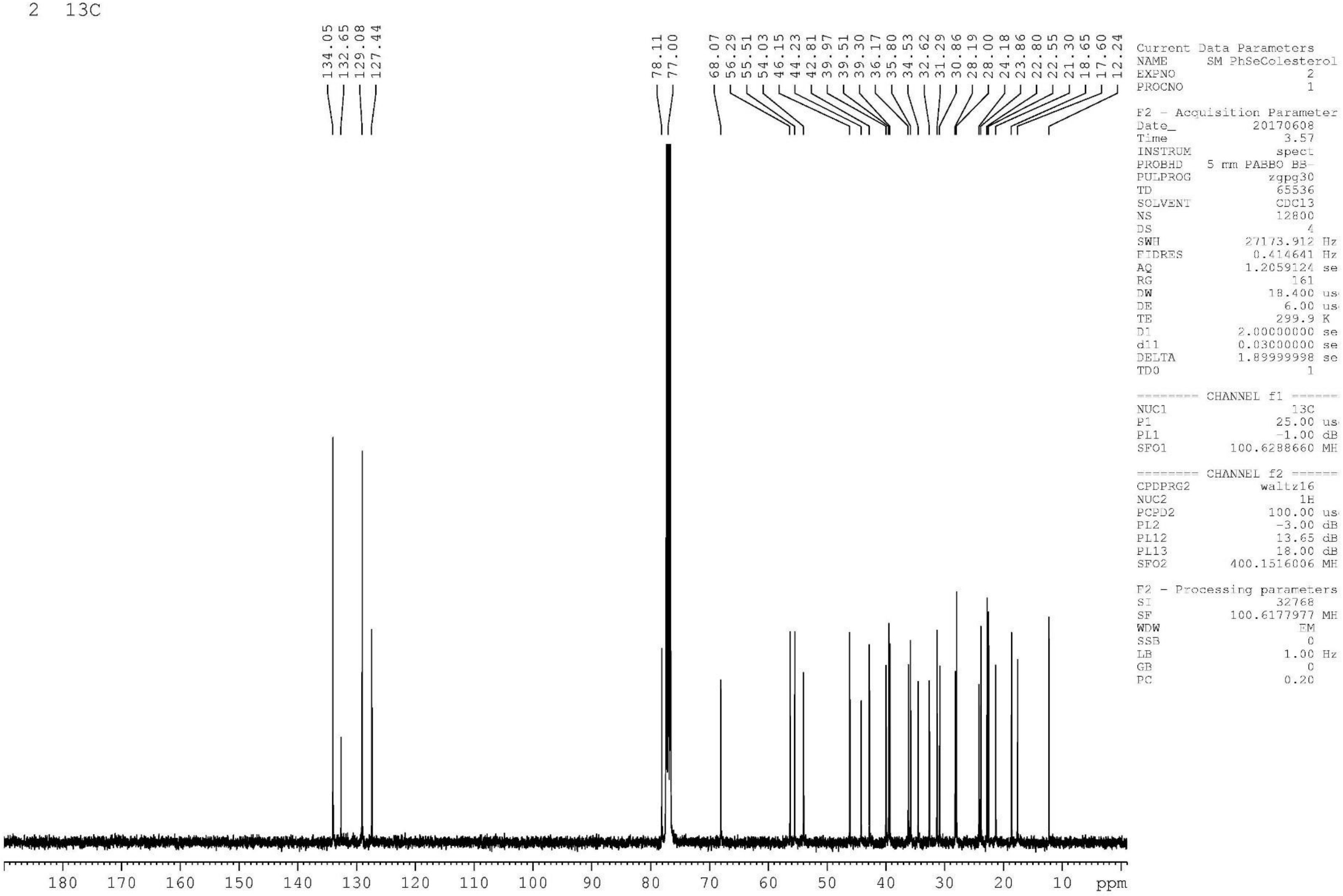

Figure S2. The ^{13}C NMR spectrum of compound **2**.

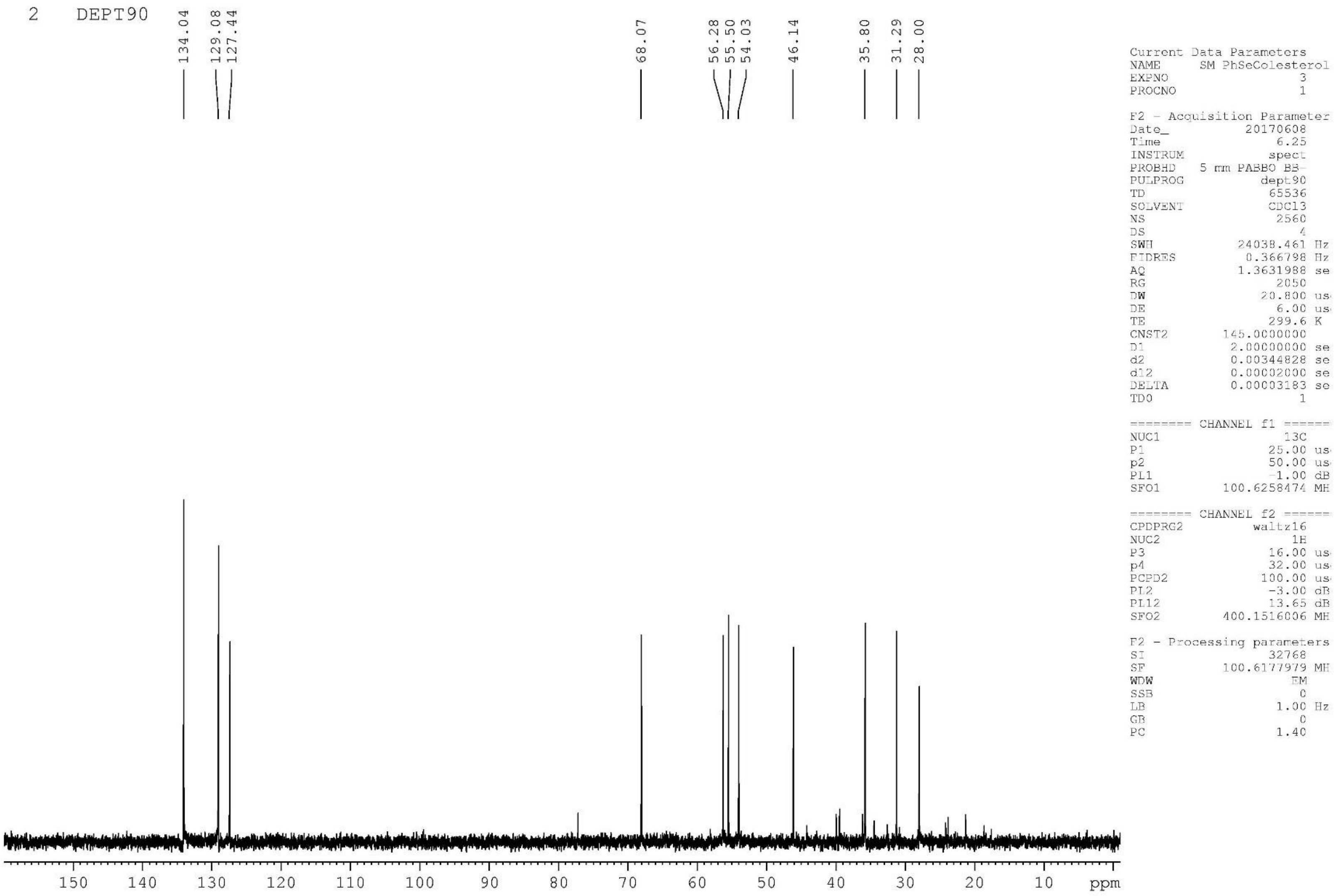

Figure S3. DEPT 90 spectrum of compound **2**.

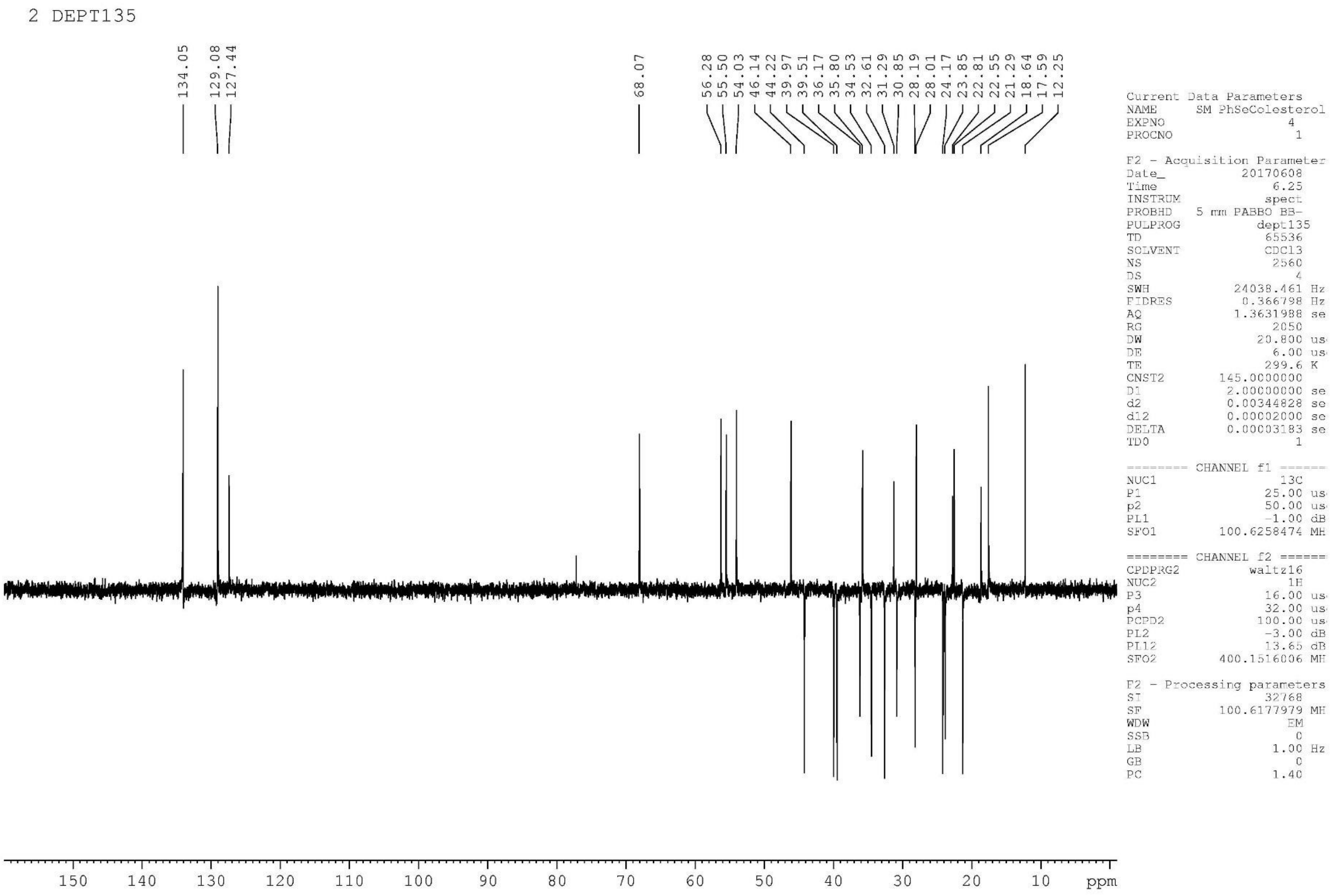

Figure S4. DEPT 135 spectrum of compound **2**.

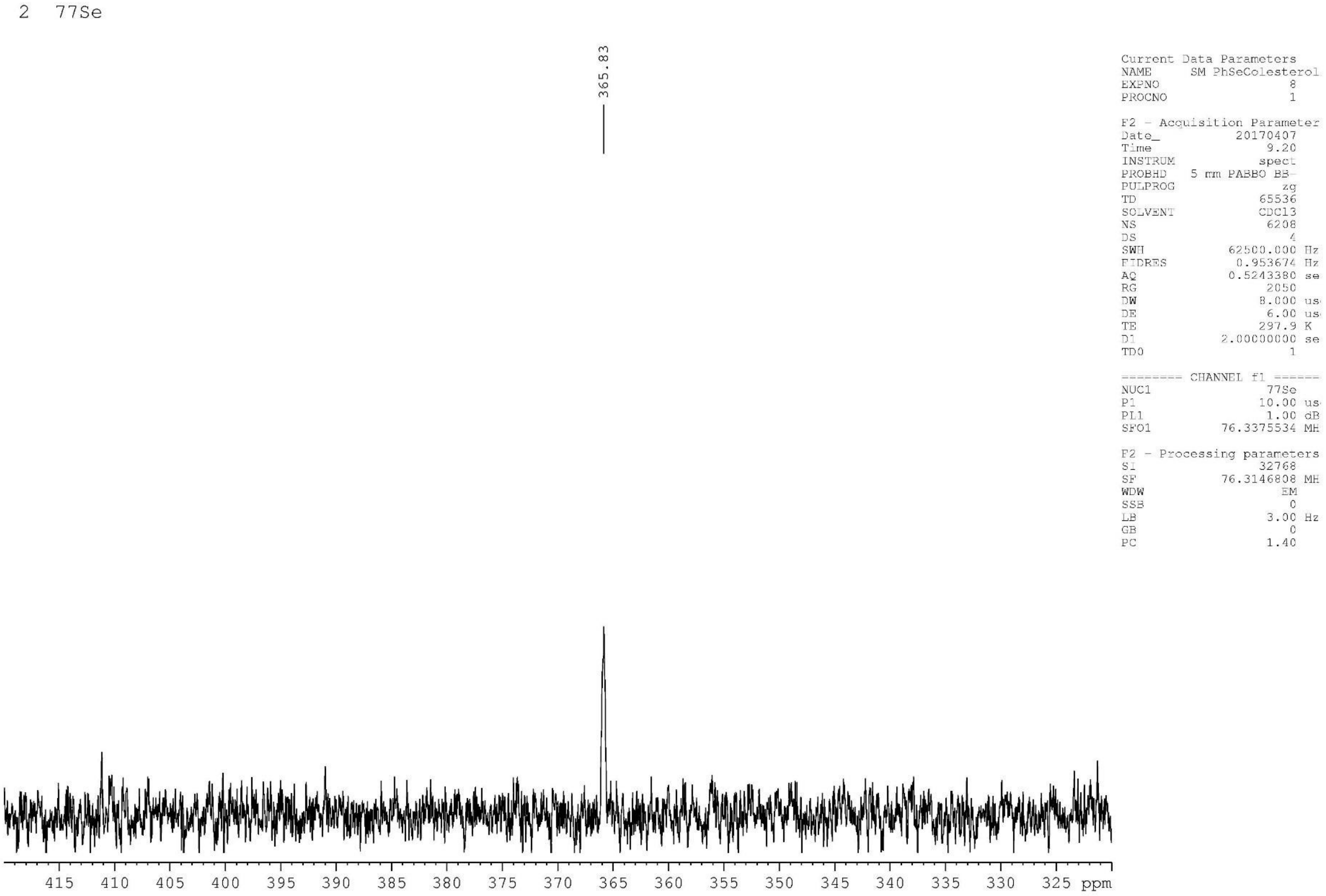

Figure S5. The ^{77}Se spectrum of compound **2**.

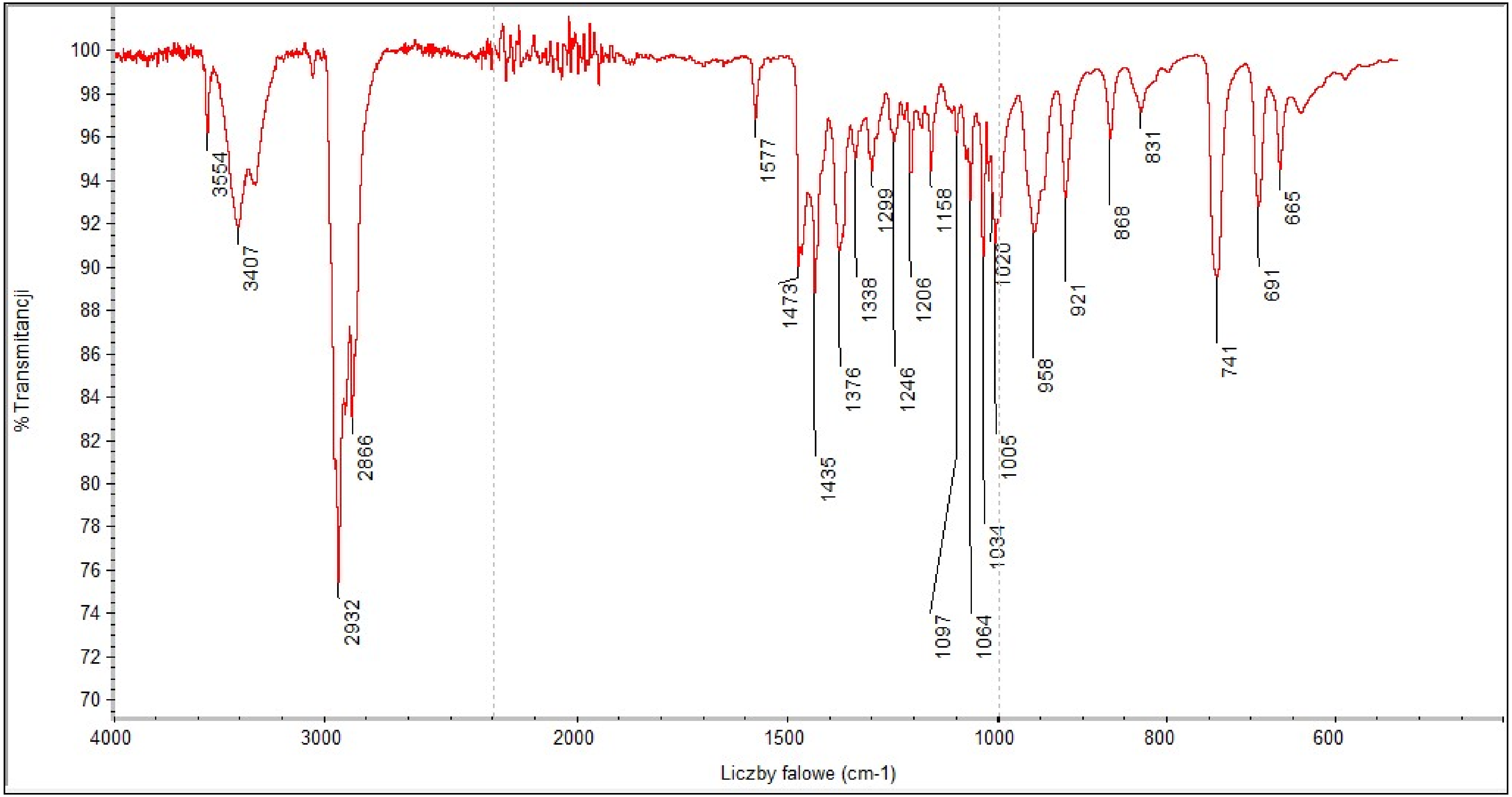

Figure S6. The IR spectrum of compound **2**.

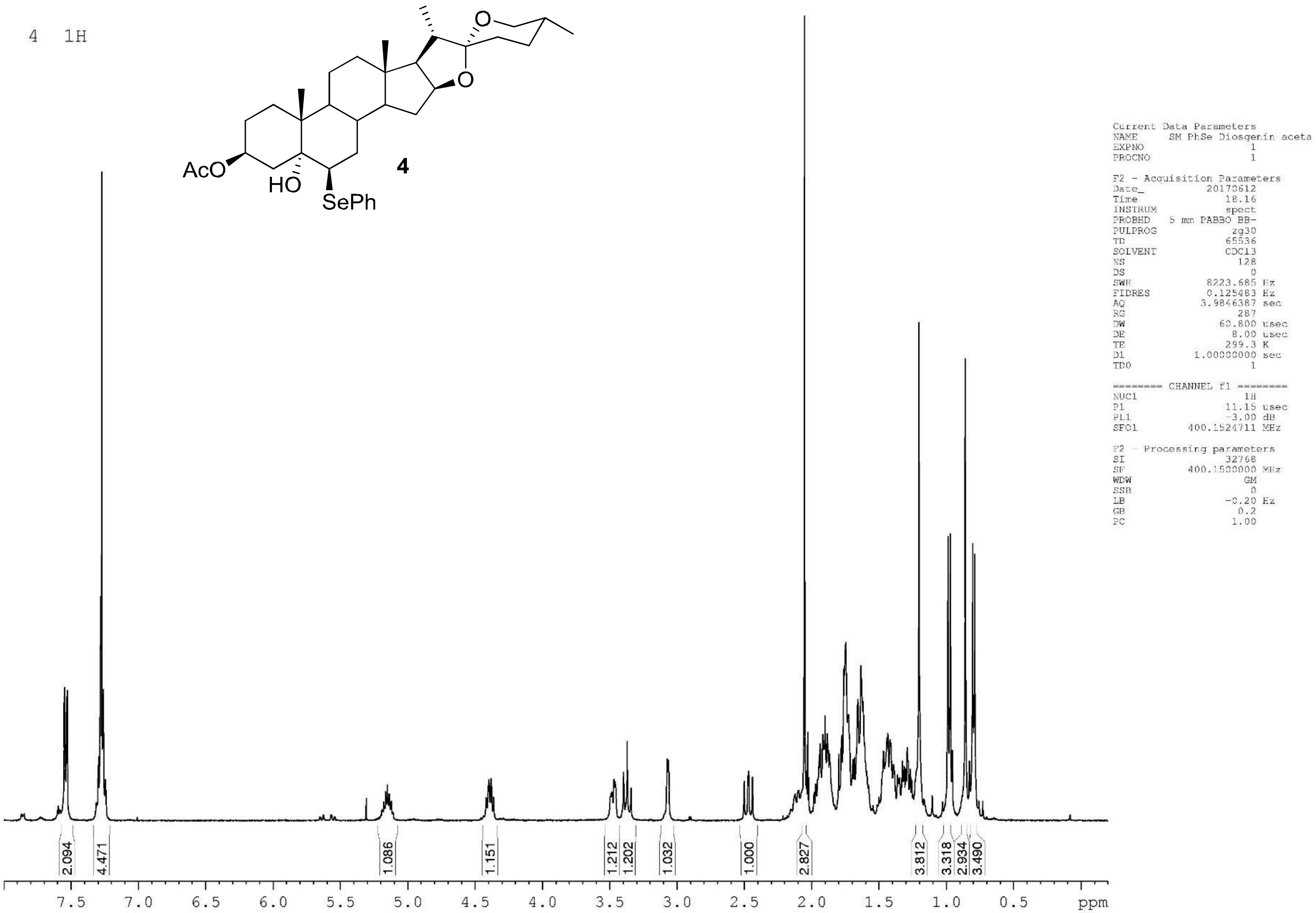

Figure S7. The ^{1}H NMR spectrum of compound **4**.

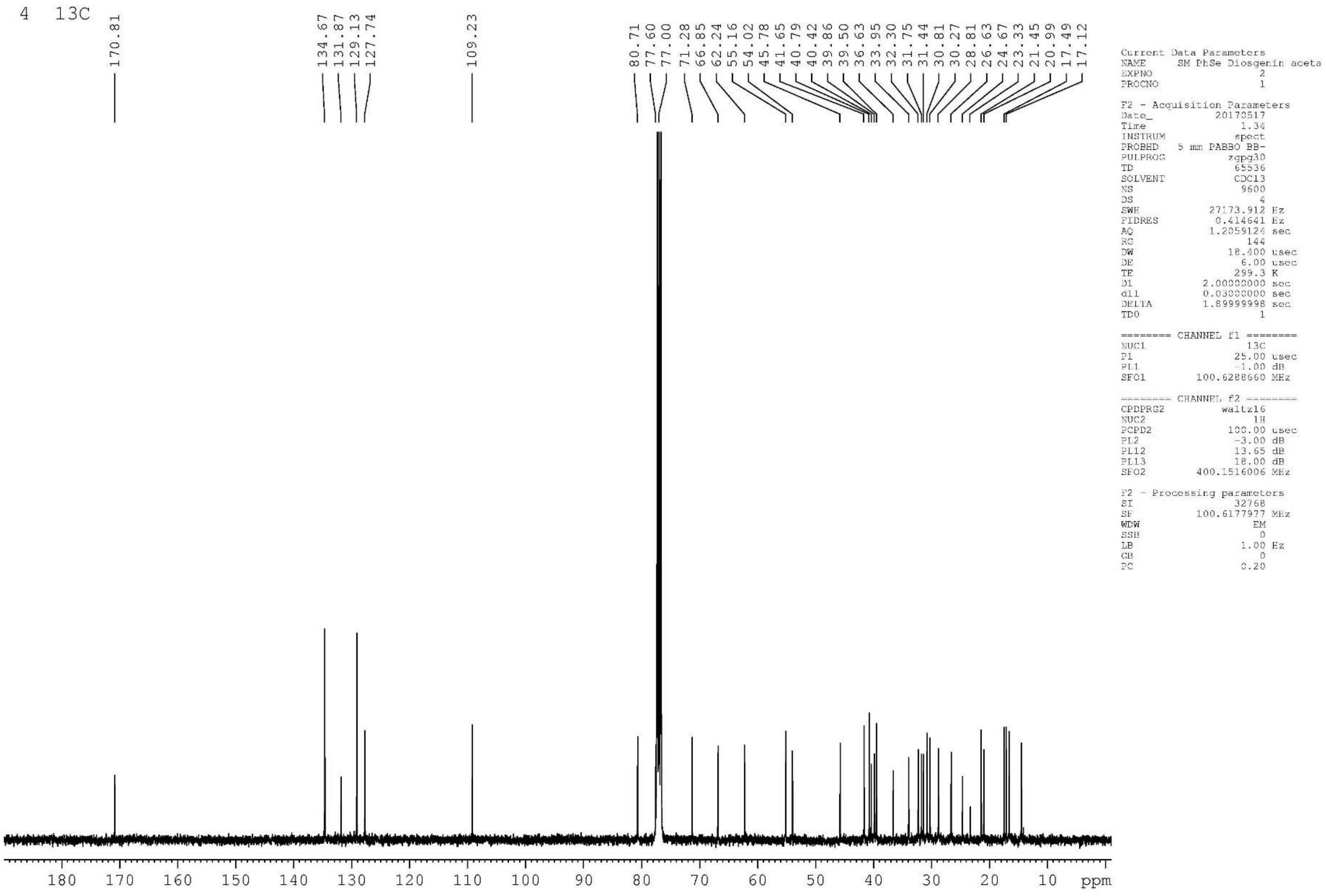

Figure S8. The ^{13}C NMR spectrum of compound **4**.

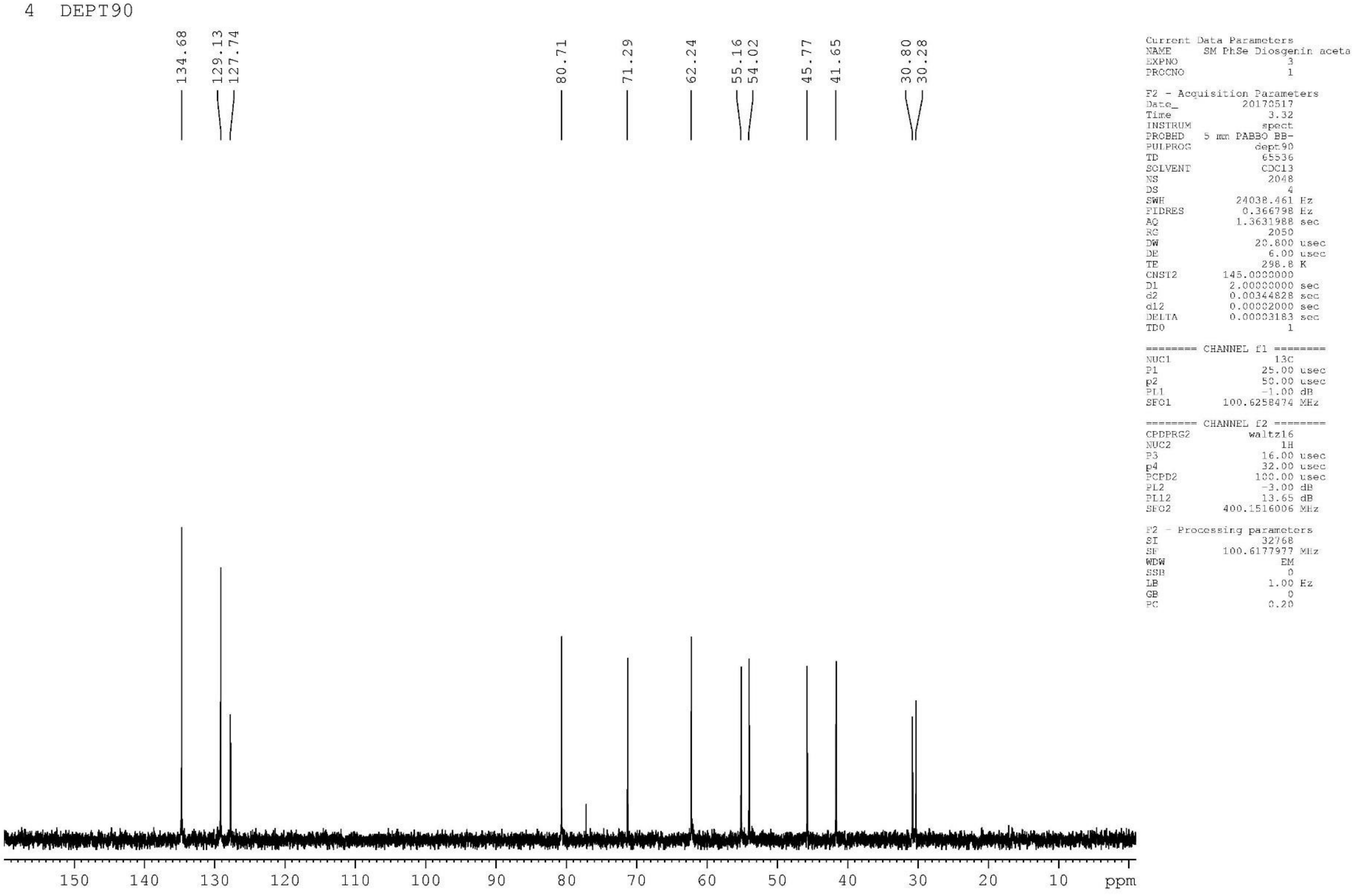

Figure S9. DEPT 90 spectrum of compound **4**.

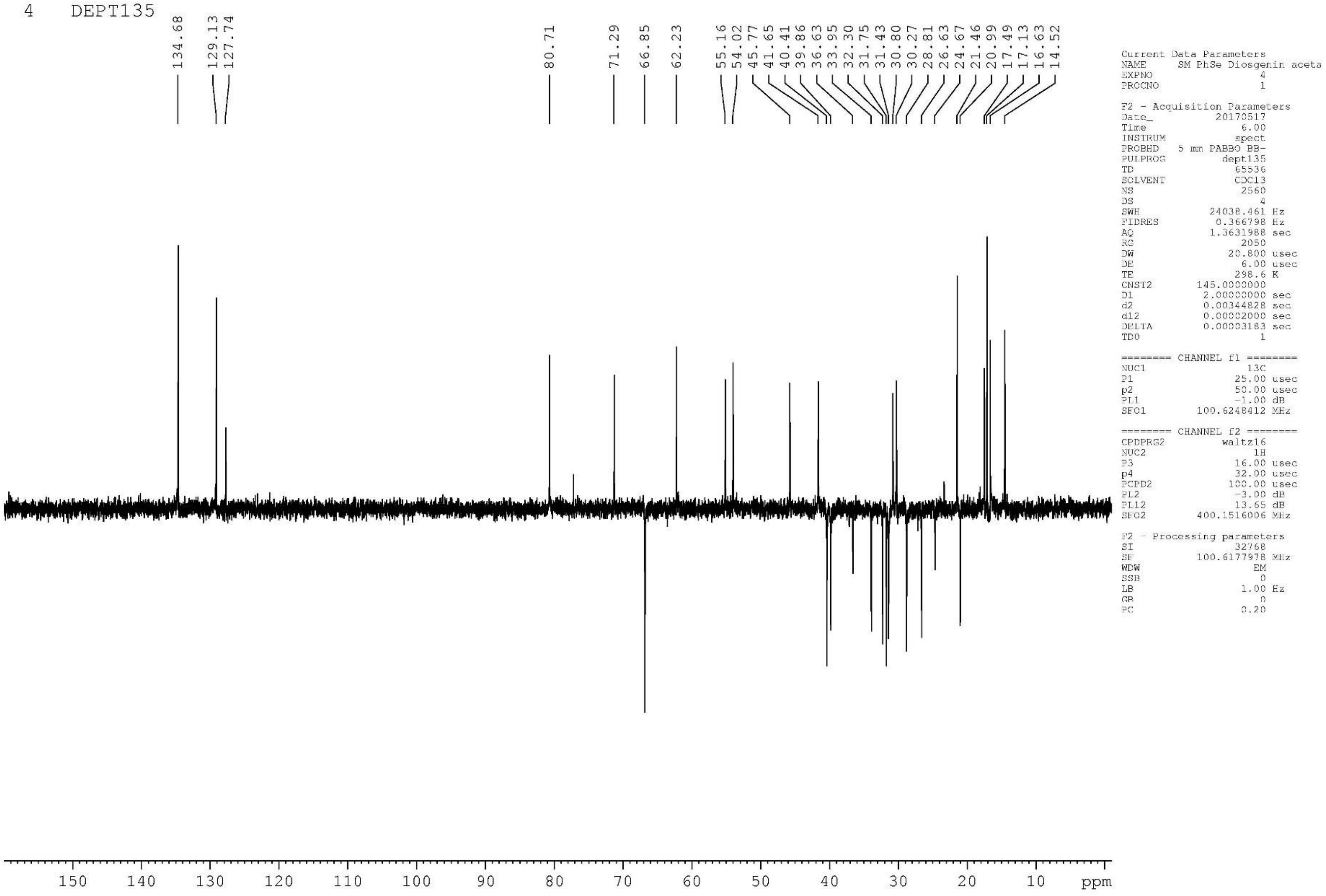

Figure S10. DEPT 135 spectrum of compound **4**.

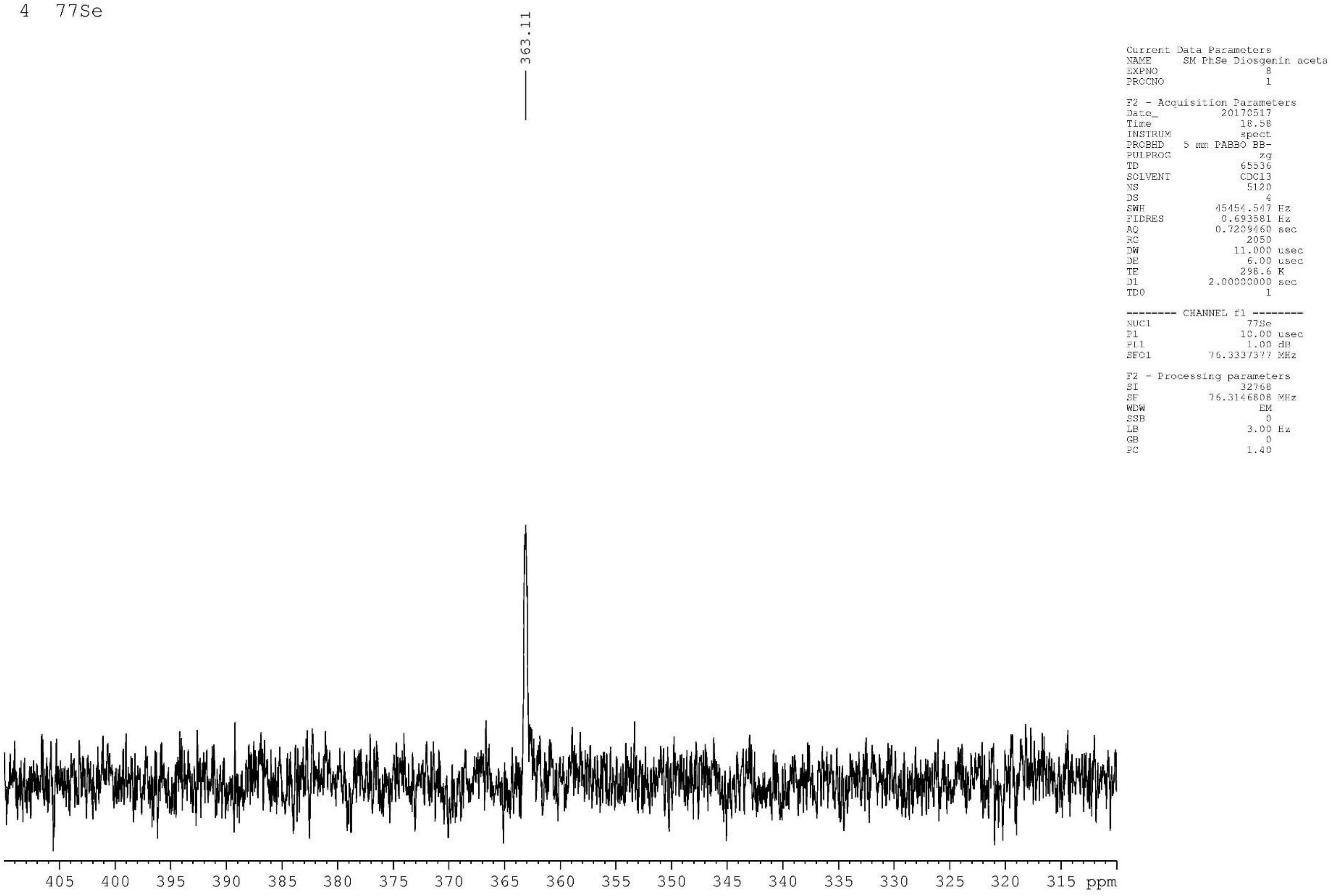

Figure S11. The ^{77}Se spectrum of compound **4**.

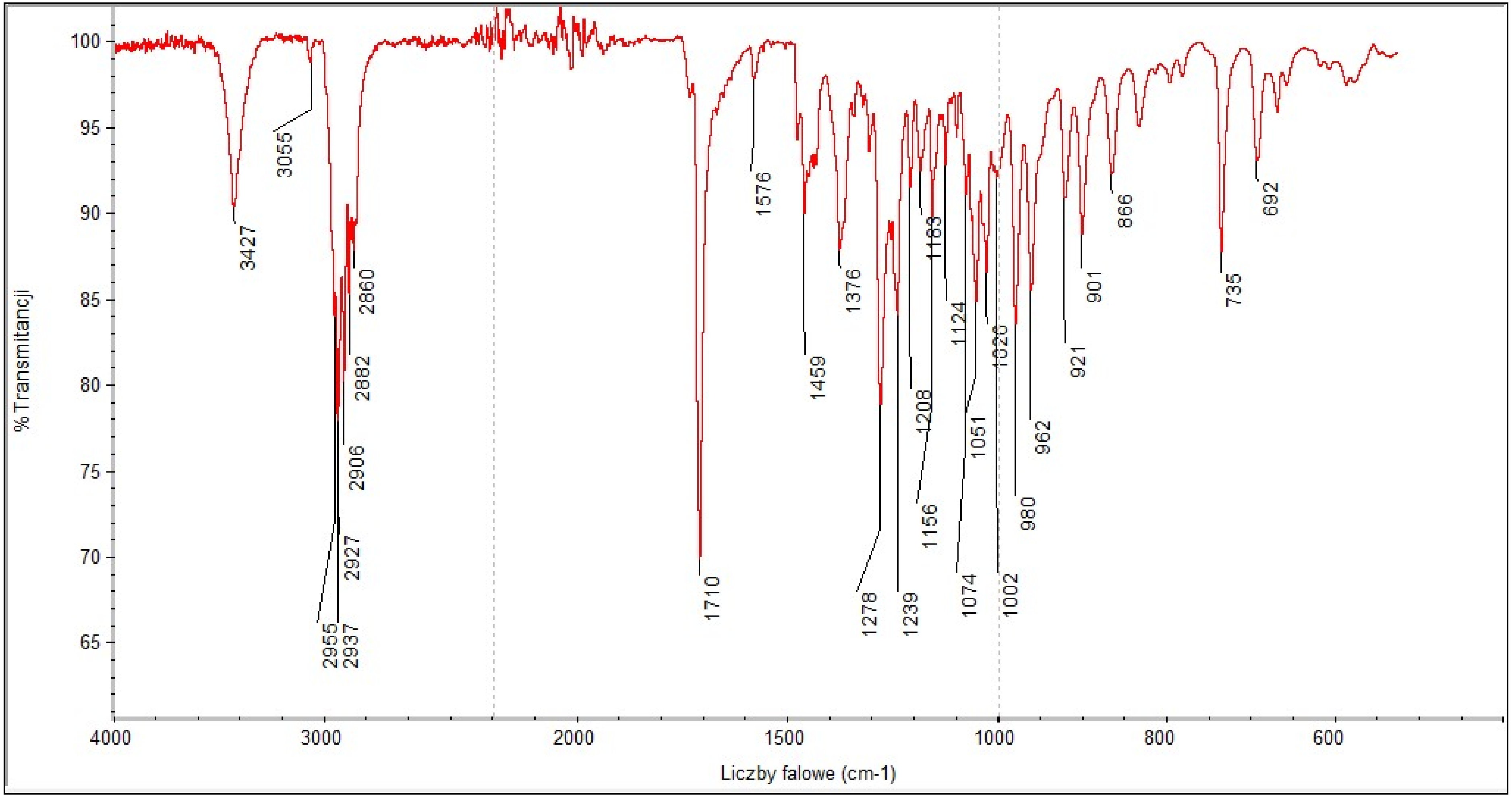

Figure S12. The IR spectrum of compound **4**.

Table S2. ^{1}H NMR and ^{13}C-NMR chemical shifts of compound **7**.

No	^{1}H [δ, ppm]	^{13}C [δ, ppm]
1	4.39 (br s)	57.41; CH
2	3.78 (m)	73.98; CH
3	4.09 (m)	67.94; CH
4	1.54, 1.37	32.96; CH_2
5	1 68	41.33; CH
6	1 33	28.67; CH_2
7	1.65, 0.88	31.47; CH_2
8	1 40	35.74; CH
9	1 16	52.43; CH
10	-	39.58; C
11	1.46, 1.37	20.73; CH_2
12	1.98, 1.16	39.57; CH_2
13	-	42.64; C
14	1 07	56.13; CH
15	1.58, 1.06	24.15; CH_2
16	1.84, 1.26	28.21; CH_2
17	1 07	56.21; CH
18	0.67 (s)	12.14; CH_3
19	0 96(s)	15.94; CH_3
20	1 43	35.08; CH
21	0.91 (d, *J* = 6.5 Hz)	18.65; CH_3
22	1.34, 1.00	36.15; CH_2
23	1.33, 1.14	23.77; CH_2
24	1 13	39.49; CH_2
25	1 52	27.99; CH

26	0.87 (d, *J* = 6.6 Hz)	22.55; CH_3
27	0.88 (d, *J* = 6.6 Hz)	22.79; CH_3
1′	-	130.21; C
2′	7 64	133.80; CH
3′	7 25	127.43; CH
4′	7.26	129.20; CH

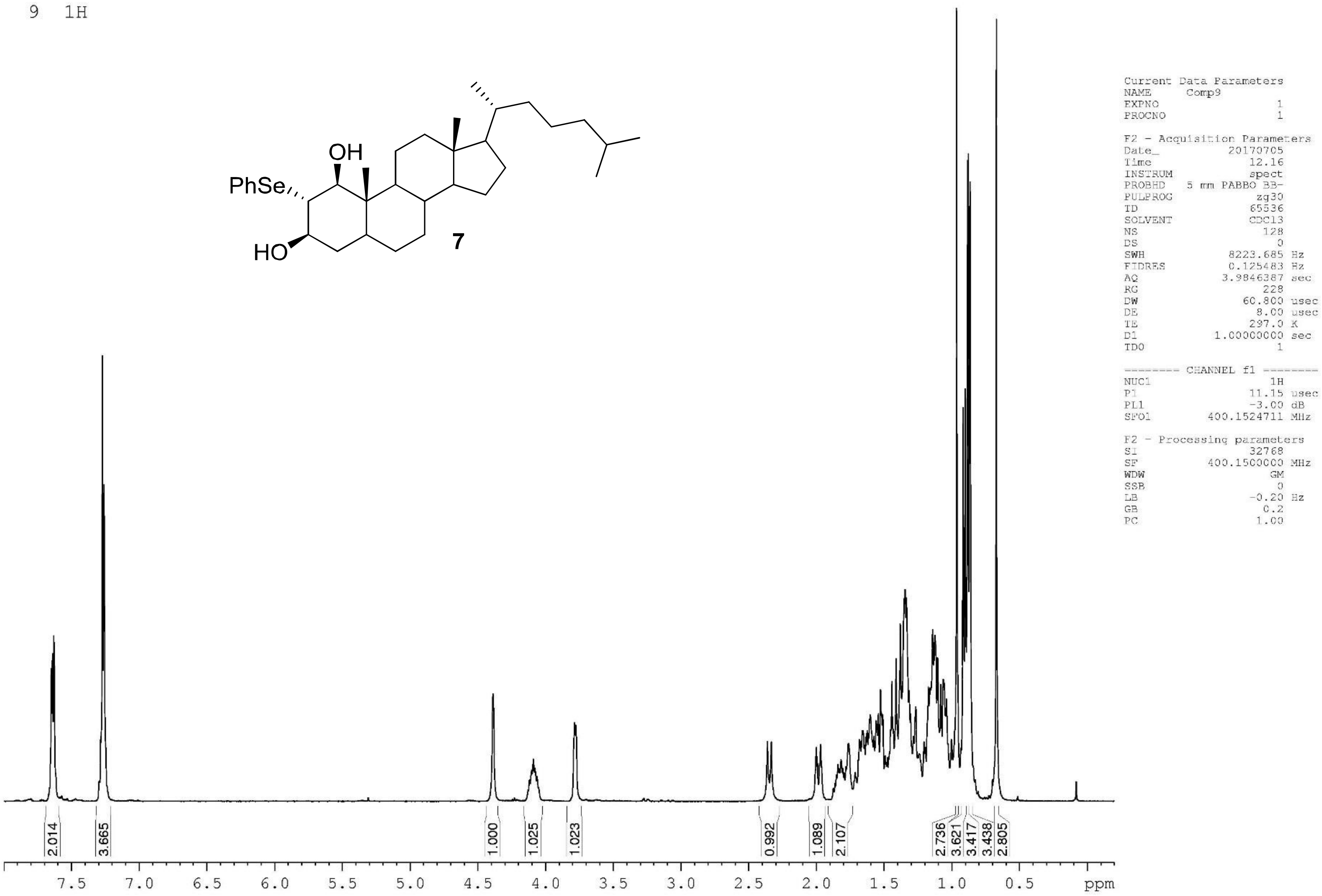

Figure S13. The ^{1}H NMR spectrum of compound **7**.

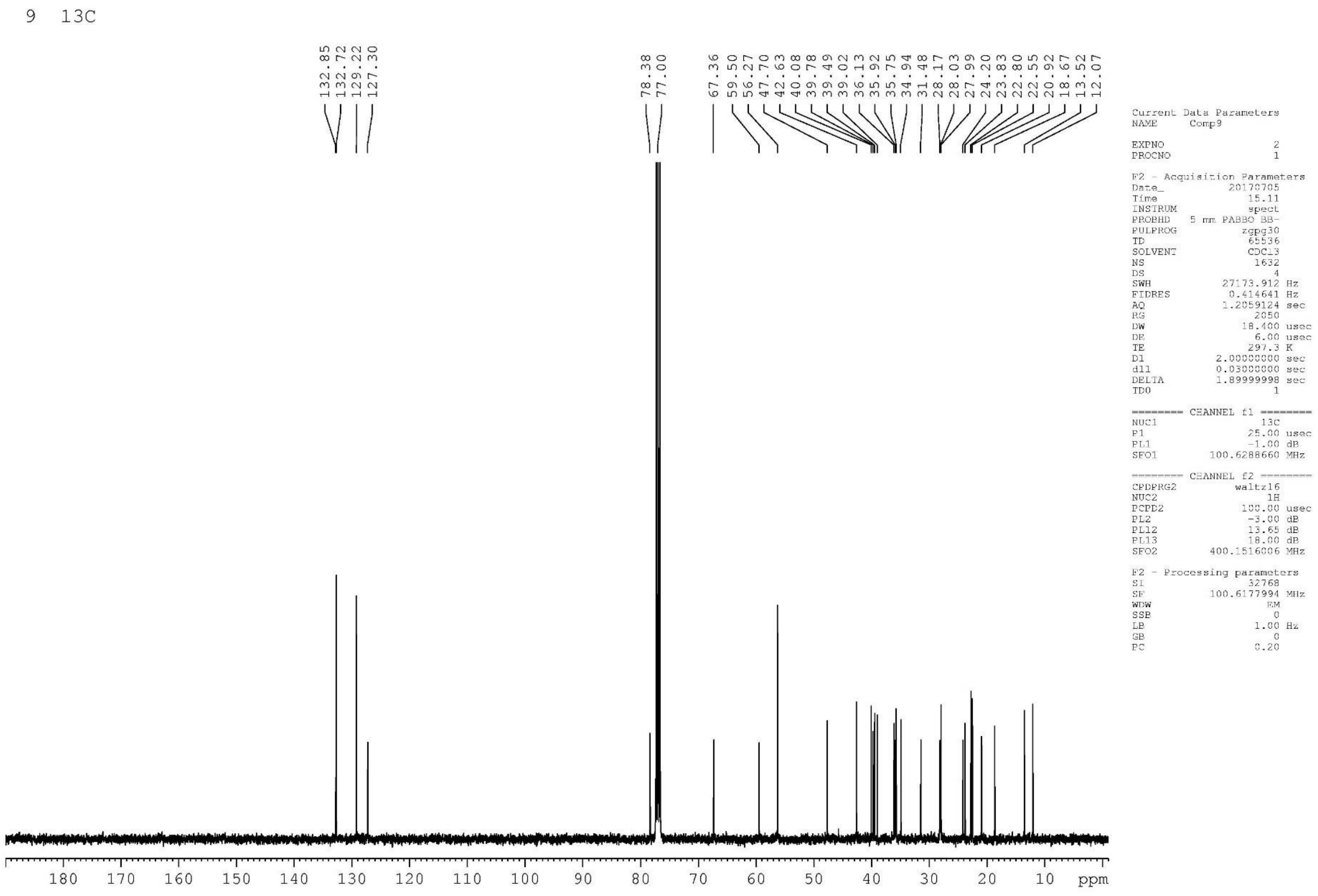

Figure S14. The ^{13}C NMR spectrum of compound **7**.

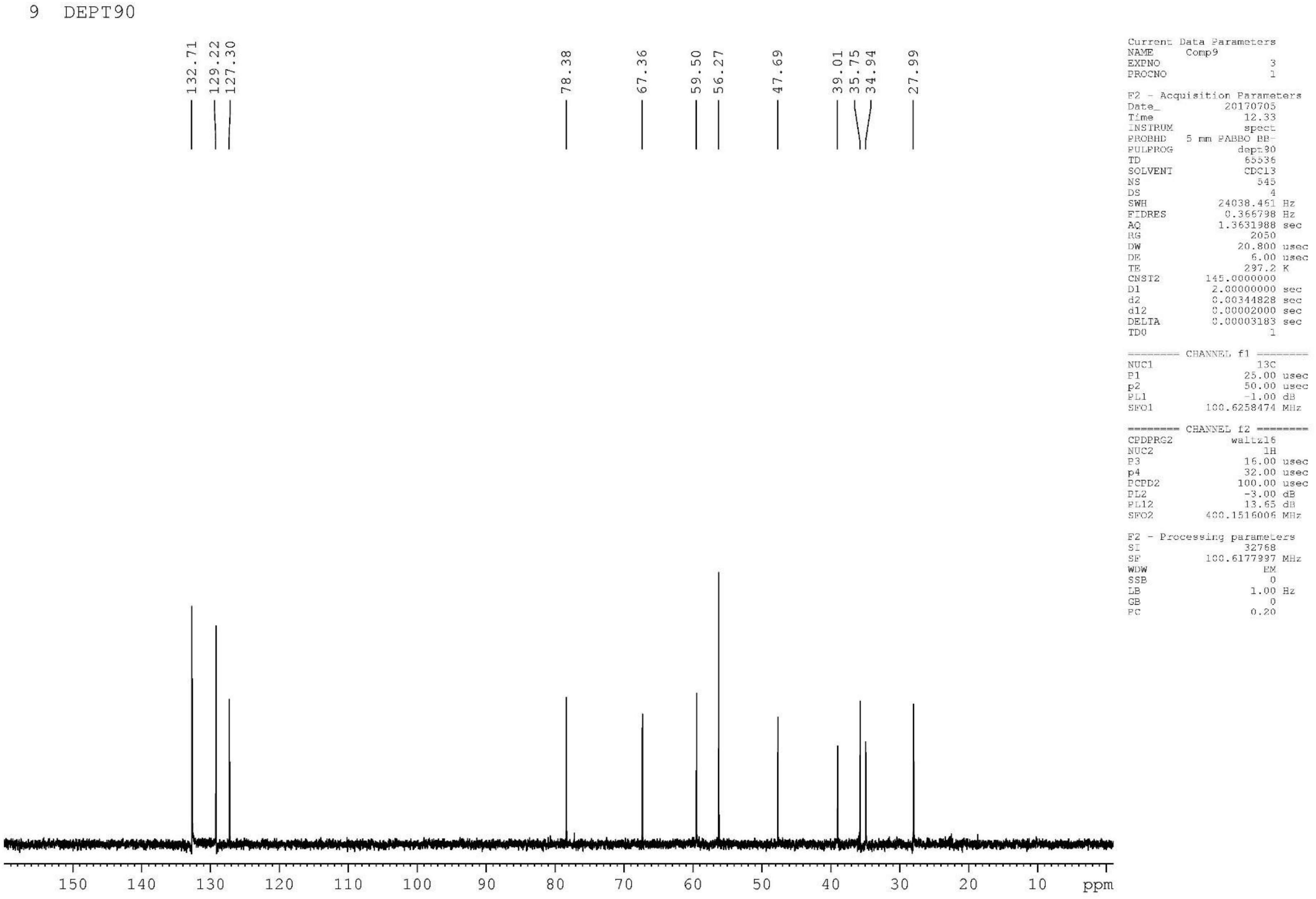

Figure S15. DEPT 90 spectrum of compound **7**.

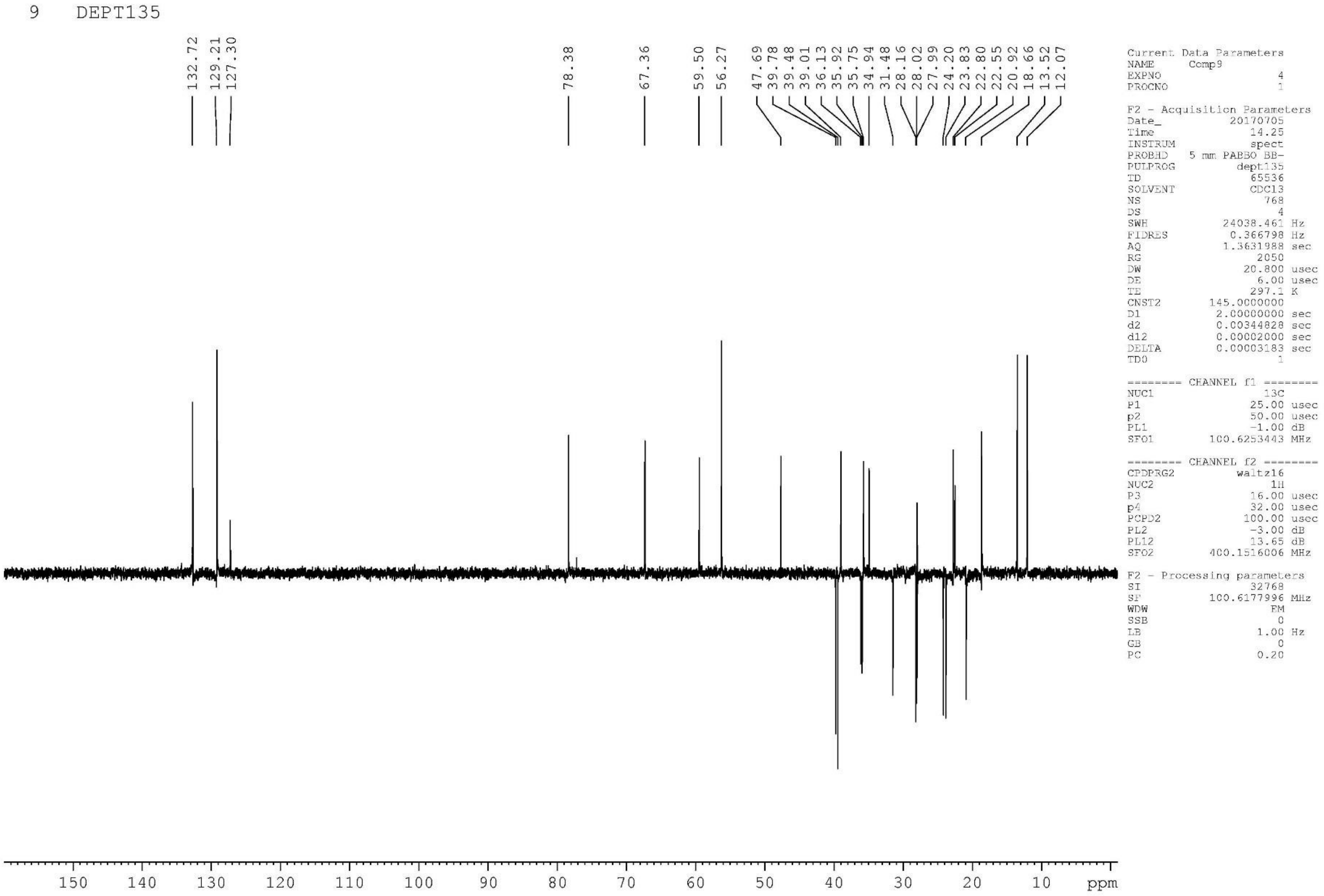

Figure S16. DEPT 135 spectrum of compound **7**.

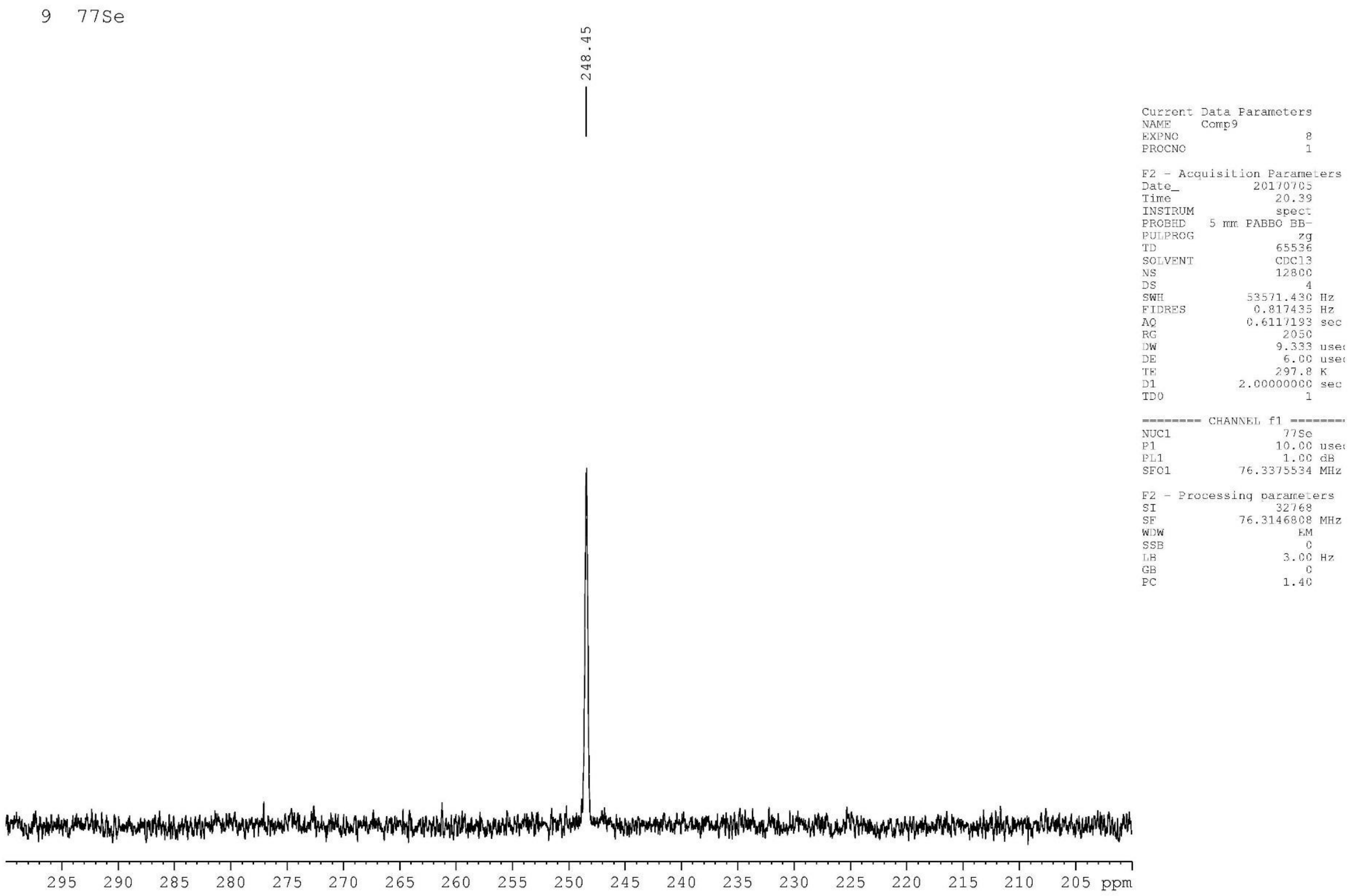

Figure S17. ^{77}Se spectrum of compound **7**

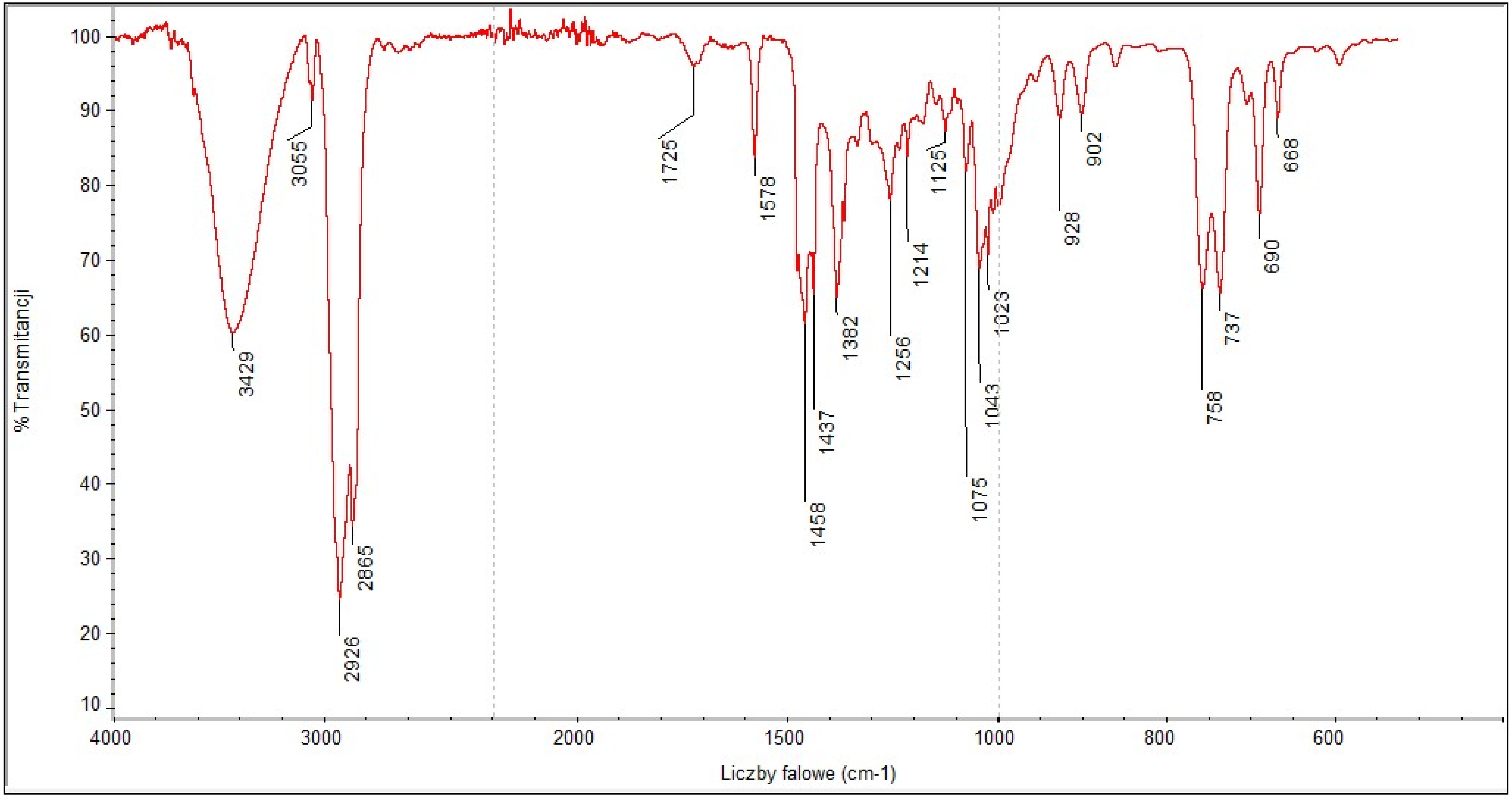

Figure S18. The IR spectrum of compound **7**.

Table S2. ^1H NMR and ^{13}C-NMR chemical shifts of compound **9**.

No	^1H [δ, ppm]	^{13}C [δ, ppm]
1	3.51 (br s)	57.41; CH
2	4.09 (m)	73.98; CH
3	4.39 (br s)	67.94; CH
4	1.62, 1.42	32.96; CH_2
5	1 41	41.33; CH
6	1 34	28.67; CH_2
7	1.967, 0.89	31.47; CH_2
8	1 38	35.74; CH
9	1 23	52.43; CH
10	-	39.58; C
11	1.66, 1.35	20.73; CH_2
12	1.98, 1.18	39.57; CH_2
13	-	42.64; C
14	1 12	56.13; CH
15	1.58, 1.06	24.15; CH_2
16	1.83, 1.25	28.21; CH_2
17	1.08.2017	56.21; CH
18	0.68 (s)	12.14; CH_3
19	1.19(s)	15.94; CH_3
20	1 43	35.08; CH
21	0.92 (d, *J* = 6.5 Hz)	18.65; CH_3
22	1.34, 1.00	36.15; CH_2
23	1.33, 1.14	23.77; CH_2
24	1 13	39.49; CH_2

25	1 52	27.99; CH
26	0.87 (d, *J* = 6.6 Hz)	22.55; CH_3
27	0.88 (d, *J* = 6.6 Hz)	22.79; CH_3
1′	-	130.21; C
2′	7 54	133.80; CH
3′	7 25	127.43; CH
4′	7.26	129.20; CH

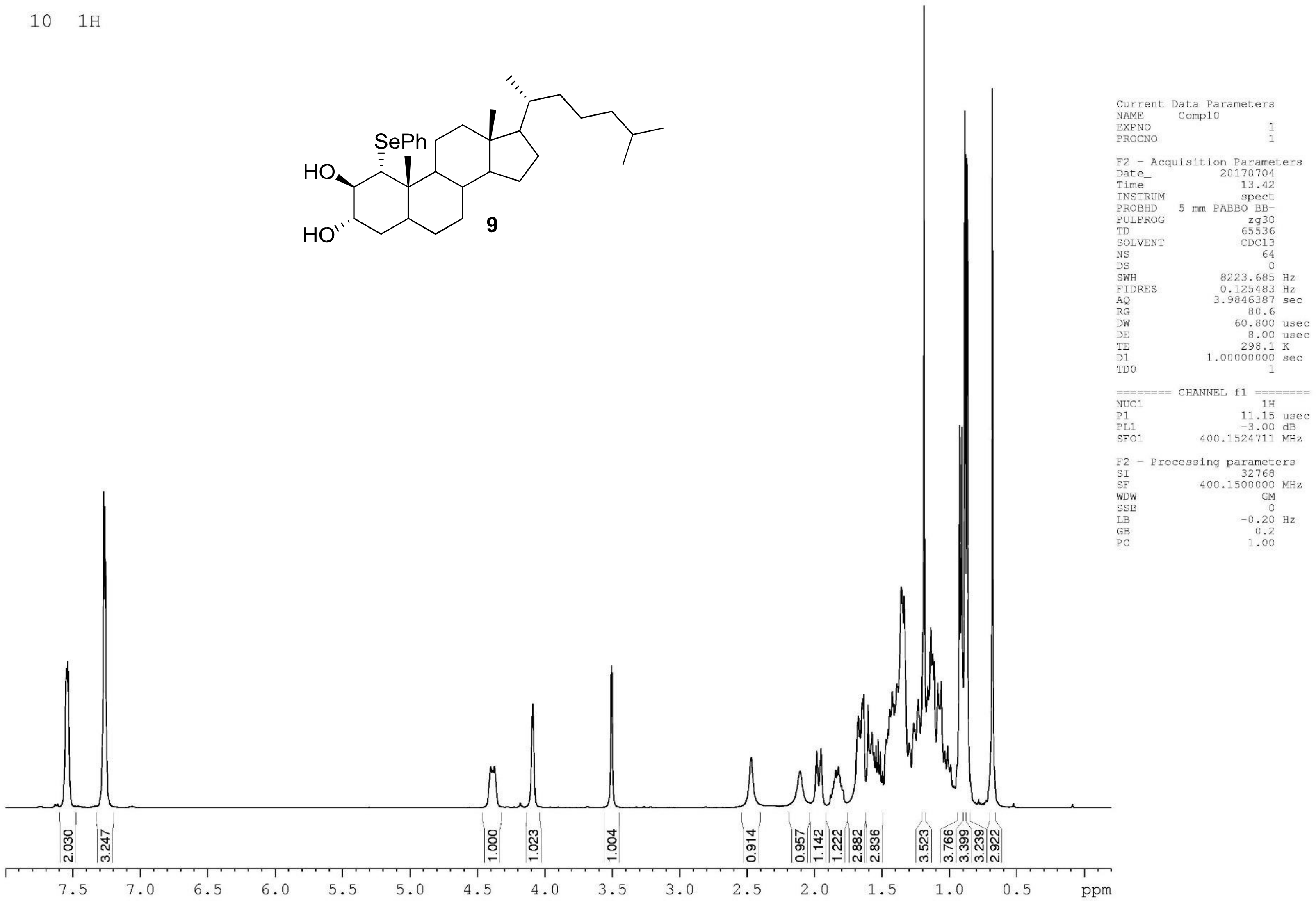

Figure S19. The ^{1}H NMR spectrum of compound **9**.

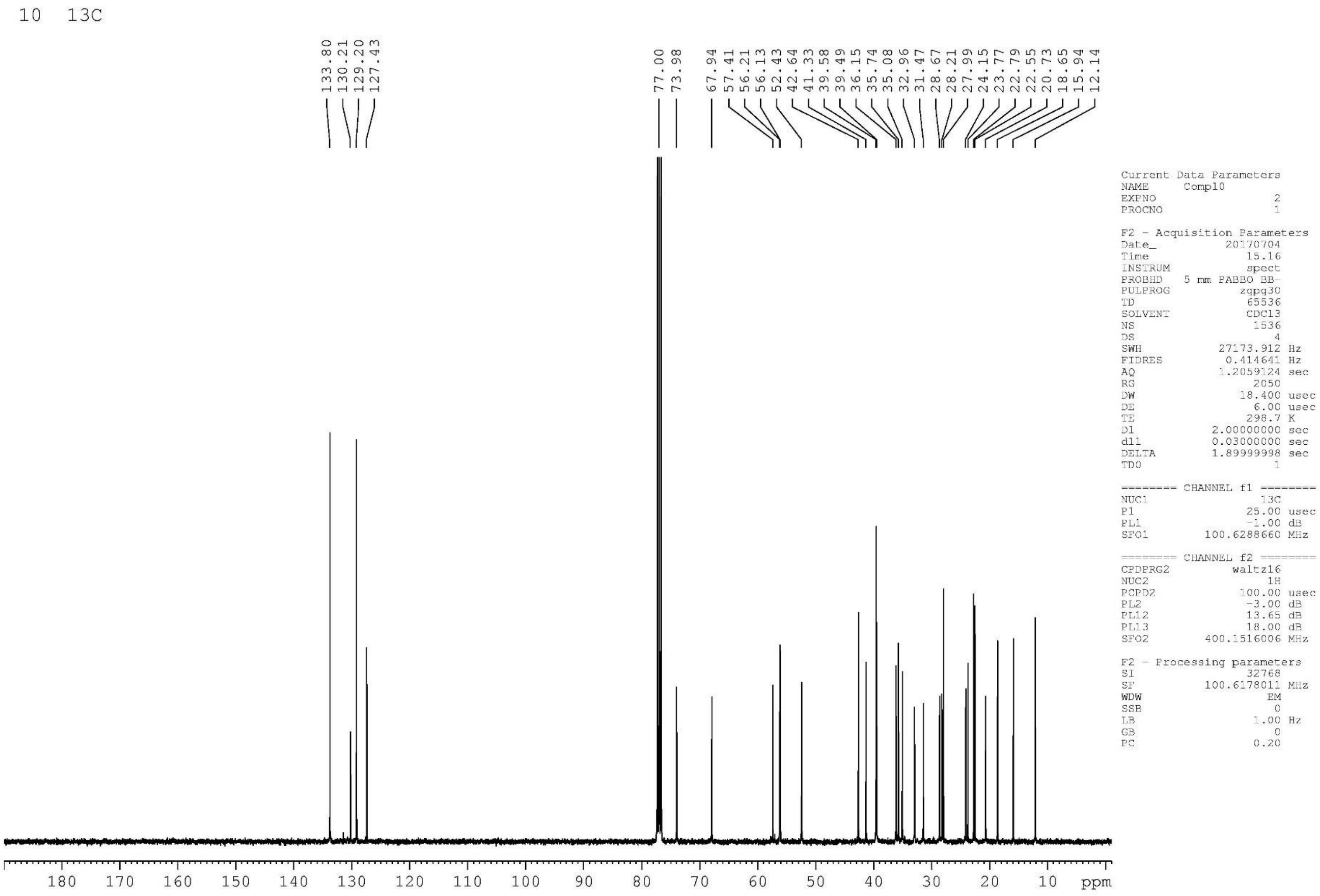

Figure S20. The ^{13}C NMR spectrum of compound **9**.

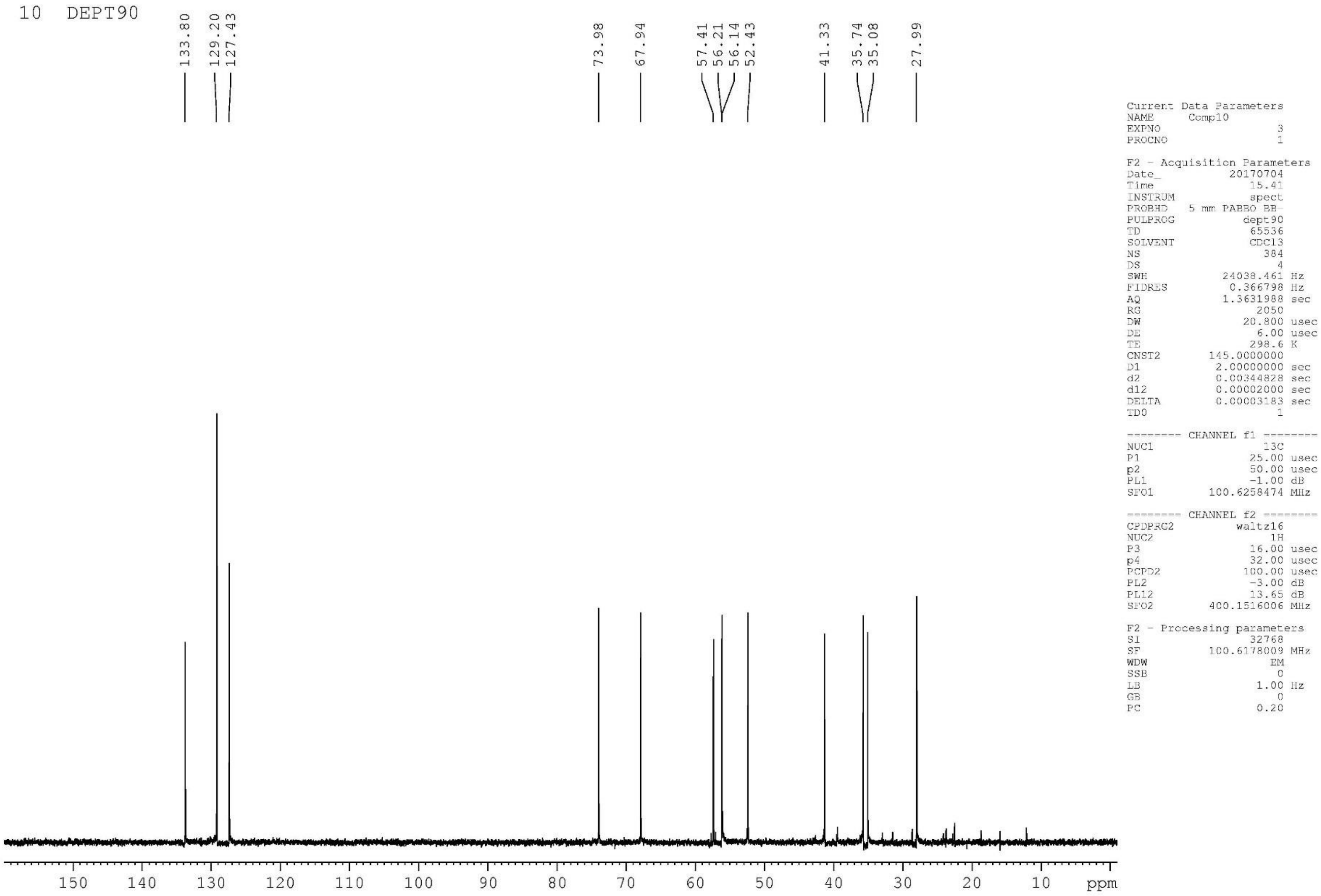

Figure S21. DEPT 90 spectrum of compound **9**.

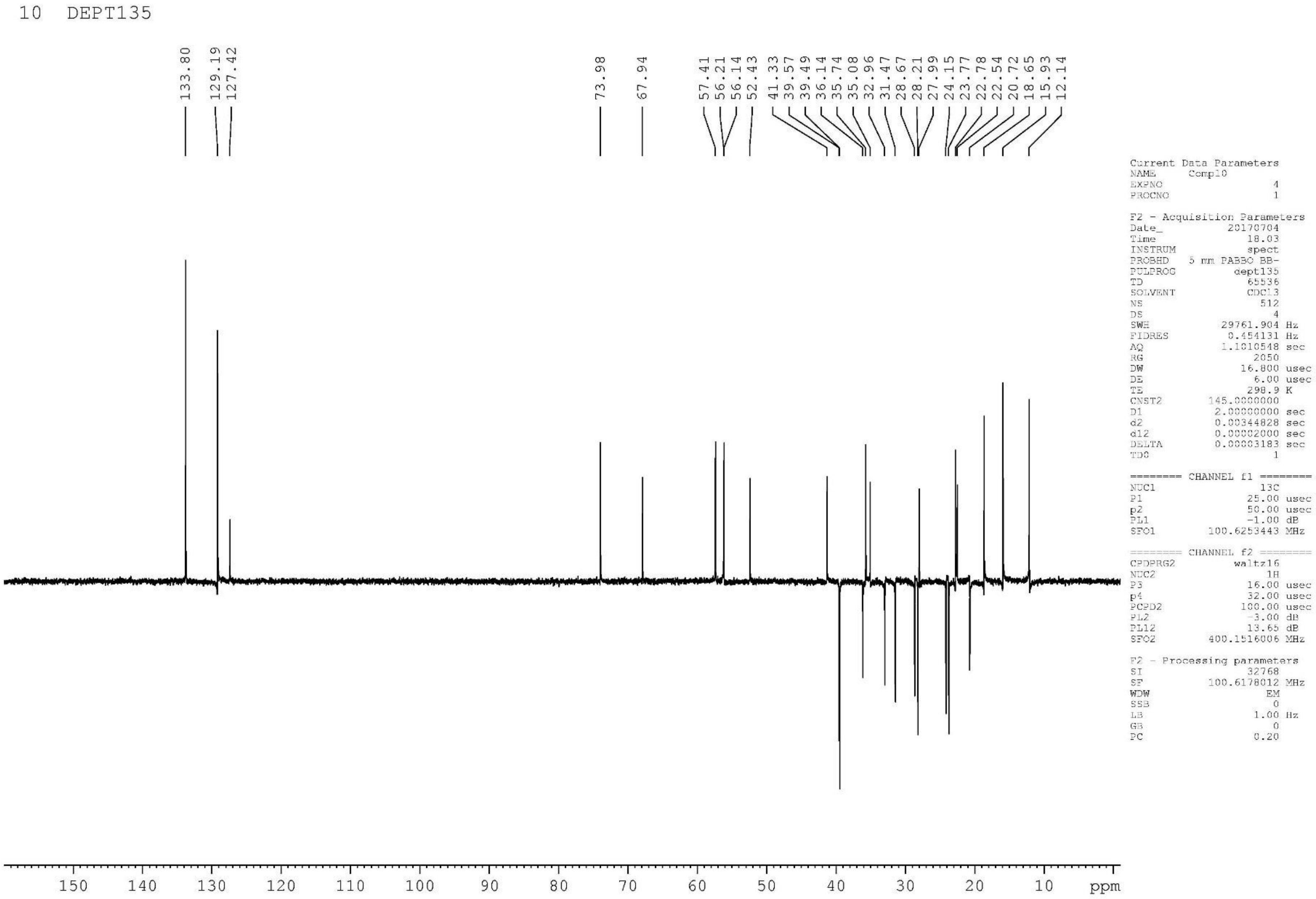

Figure S22. DEPT 135 spectrum of compound **9**.

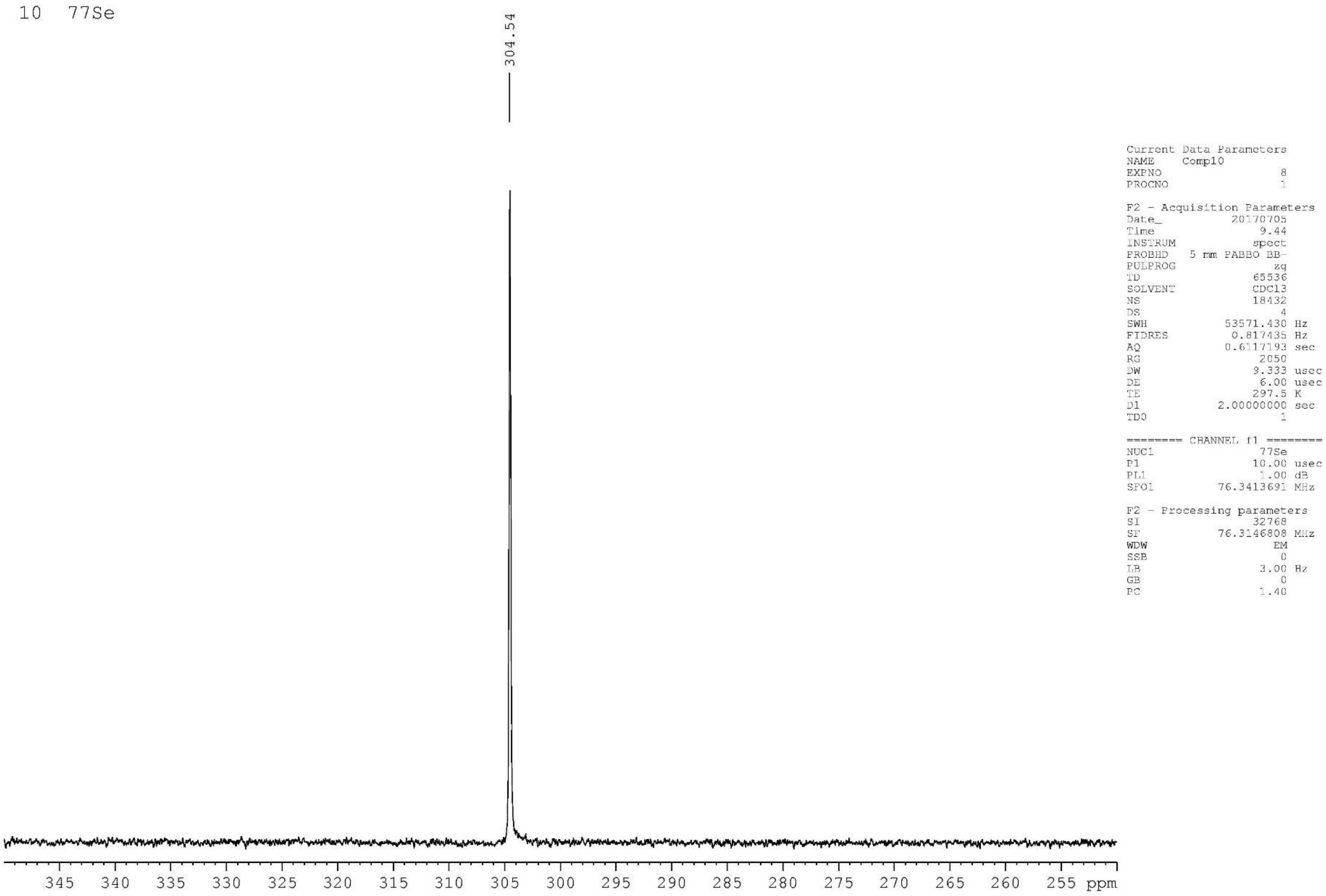

Figure S23. The ^{77}Se spectrum of compound **9**.

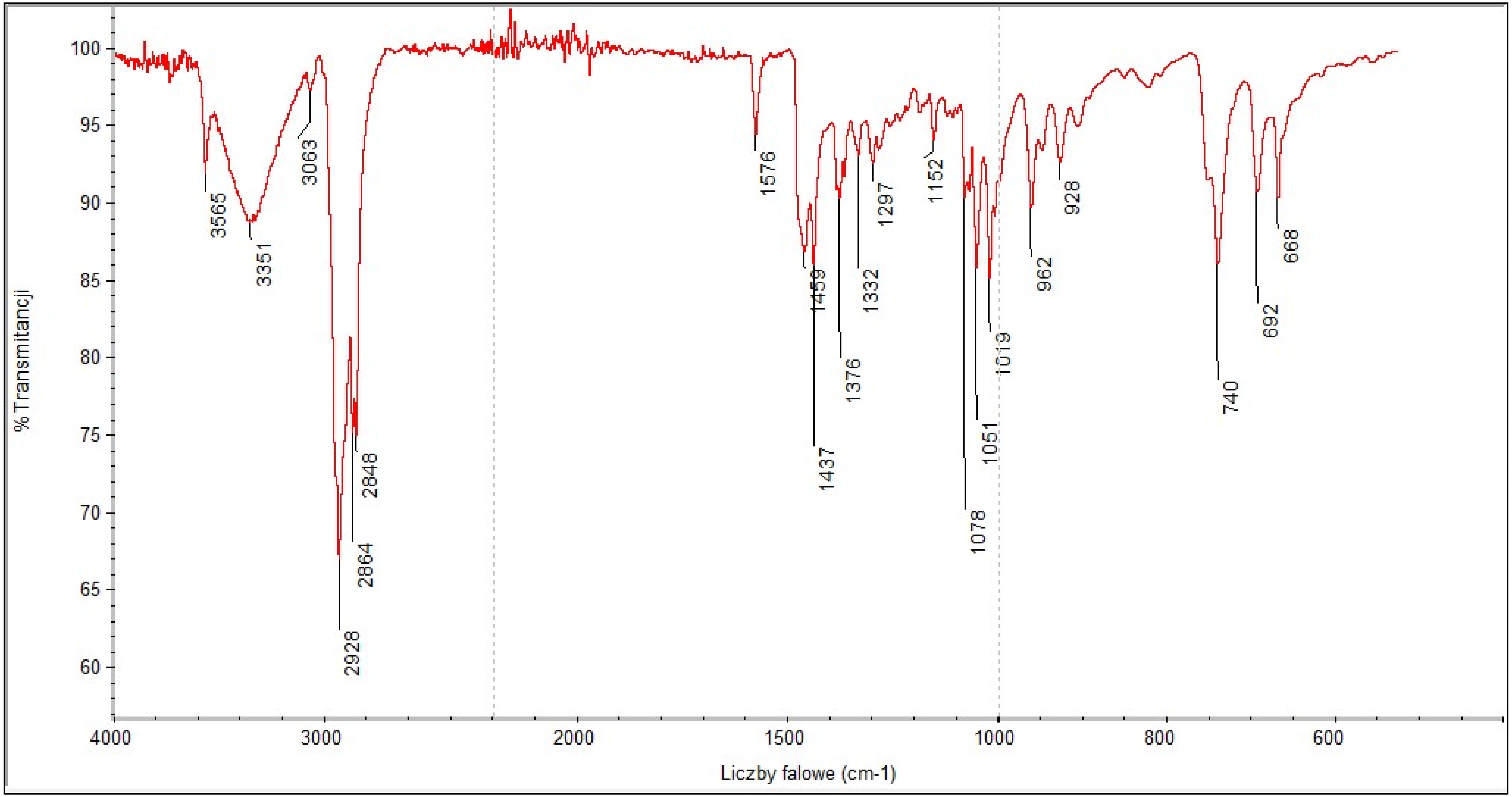

Figure S24. The IR spectrum of compound **9**.
